# Burden of disease study of overweight and obesity; the societal impact in terms of cost-of-illness and health-related quality of life

**DOI:** 10.1186/s12889-021-12449-2

**Published:** 2022-01-07

**Authors:** J. Hecker, K. Freijer, M. Hiligsmann, S. M. A. A. Evers

**Affiliations:** 1 Department of Radiology and Nuclear Medicine, Maastricht University Medical Center, Maastricht, The Netherlands; 2 Department of Health Service Research, Care and Public Health Research Institute (CAPHRI), Maastricht University, PO box 616, 6200 MD Maastricht, The Netherlands; 3 Partnerschap Overgewicht Nederland (PON), Erasmus MC, Rotterdam, The Netherlands; 4 Centre for Economic Evaluation and Machine learning, Netherlands Institute of Mental Health and Addiction, Trimbos Institute, Utrecht, The Netherlands

**Keywords:** Overweight, Obesity, Dutch population, Cost of illness, Burden of disease, Health-related quality of life

## Abstract

**Background:**

Little is known about the burden that overweight and obesity impose on Dutch society. The aim of this study is to examine this burden in terms of cost-of-illness and health-related quality of life.

**Method:**

A bottom-up, prevalence-based burden of disease study from a societal perspective was performed. Cost-of-illness information including healthcare costs, patient and family costs, and other costs was obtained via the Treatment Inventory of Costs in Patients with psychiatric disorders (TiC-P) questionnaire. Health-related quality of life was assessed through the EuroQol (EQ-5D-5L) and the BODY-Q instruments. Non-parametric bootstrapping was applied to test for significant differences in costs. Subgroup analyses were performed on all outcomes.

**Results:**

A total of 97 people with overweight and obesity completed the survey. Per respondent, mean healthcare costs were €2907, patient and family costs were €4037, and other costs were €4519, leading to a total societal cost of €11,463 per respondent per year. Total costs were significantly higher for respondents with obesity versus overweight and between low & intermediate versus highly educated respondents. The mean utility score of our population was 0.81. A significantly lower utility score was found for respondents with obesity in comparison with respondents with overweight. BODY-Q results show that respondents with obesity scored a significantly lower Rasch-score than did respondents with overweight in three scales. Respondents with a high education level and having paid work scored significantly higher Rasch-scores in two scales than did those with a low education level and without having paid work. The age group 19–29 have significantly higher Rasch-scores in three scales than respondents in the other two age categories.

**Conclusions:**

Overweight and obesity have a considerable impact on the societal costs and on health-related quality of life. The results show that the impact of overweight and obesity go beyond the healthcare sector, as the other costs have the biggest share of the total costs. Another interesting finding of this study is that obesity leads to significant higher costs and lower health-related quality of life than overweight. These findings draw attention to policy making, as collective prevention and effective treatment are needed to reduce this burden.

**Supplementary Information:**

The online version contains supplementary material available at 10.1186/s12889-021-12449-2.

## Background

Globally, the prevalence of overweight (defined as body mass index (BMI) ≥ 25 kg/m^2^) and obesity (defined as BMI ≥ 30 kg/m^2^) among adults aged 18 years and older has been rising over the past few decades [1]. Between 1975 and 2016 the prevalence of obesity has nearly tripled worldwide [1]. In 2016 there were 1.9 billion adults with overweight; of these, 650 million adults were suffering from obesity [1]. In 2020, 50% of Dutch adults were overweight, of whom 13.9% were suffering from obesity [2]. The American Medical Association (AMA), the European Centre for Disease Prevention and Control (ECDC), and the European Commission have recognized obesity as a non-communicable disease with several pathophysiological aspects, such as diabetes mellitus and hypertension. These aspects require a range of interventions to advance the treatment and prevention of obesity [3, 4]. Obesity can be ranked in multiple groups; a BMI between 30.00 and 34.99 kg/m^2^ is stated as obesity class one, a BMI between 35.00 and 39.99 kg/m^2^ is obesity class 2 and BMI > 40.00 kg/m^2^ is obesity class 3 [5]. Several studies have shown that obesity and overweight are a public health problem, as it is a risk factor for several health issues. First, obesity and overweight can cause physical problems, such as coronary heart disease, diabetes type 2, hypertension and stroke, certain types of cancer, and pulmonary diseases [1, 6]. Secondly, and equally important, obesity and overweight can cause psychological problems, such as depression, stress and anxiety [7, 8]. In addition, obesity increases the risk of severe illness or death from the COVID-19 virus [9]. Moreover, obesity causes societal and economic burdens. The unhealthy years due to sickness and limitations as a result of obesity have a rising impact on societal costs. These include healthcare costs, patient and family costs, and other costs, such as productivity losses [10–12]. According to Neovius et al., (2012) productivity losses are almost twice as high for people with obesity in comparison with people with healthy weight (defined as BMI > 18.5 and < 25 kg/m^2^) over a lifetime. Furthermore, research from the Organization for Economic Cooperation and Development (OECD) shows an estimated cost of 172 Euros per capita for treating high BMI (≥ 25 kg/m^2^) and associated conditions in the OECD countries [13]. In addition, available data from multiple countries show that the costs attributable to obesity represent 5.5 to 7.8% of total healthcare expenditures [14]. Due to the physical and psychological problems, people with obesity are, among other things, hampered in their capacity to perform their daily activities, which has a devastating impact on their health-related quality of life (HRQoL) [15]. Other studies show that there is a relation between weight loss and improved HRQoL; one main reason for this relation is the reduction of metabolic co-morbidities associated with weight loss, such as diabetes mellitus, hypertension, and cardiovascular disease [16–18].

Despite the international studies showing that overweight and obesity have significant impact on the individual, the healthcare system and the society, there is no study in the Netherlands that reflects the total burden, including costs and HRQoL, that obesity and overweight have on the society as a whole. Furthermore, there is no study in the Netherlands that makes a comparison between overweight and obesity. Knowledge about these actual costs and the associated burden is needed to highlight the importance of the problem for policy and research agendas, and thereby stimulate collective prevention and treatment programs [19]. The aim of this study is to examine the societal burden of overweight and obesity on the Dutch population in terms of cost-of-illness (COI) and HRQoL.

## Methods

### Study design and setting

This is a prevalence based, bottom-up, prospective study focusing on the burden of disease expressed in COI (Euros) and HRQoL (utilities and Rasch-scores) from a societal perspective, overall based on the Dutch guidelines for costing studies in the healthcare sector [20]. The societal perspective is the preferred perspective in health economic evaluation, such as burden of disease [21, 22]. The societal perspective means that analyst considers all costs and effects that flow from the intervention, regardless who experiences these [23].

When information was not present in the Dutch guidelines, such as cost information, other sources were used. A numerical code was assigned to each participant as identification, to ensure anonymity of the questionnaire. The results obtained were available only to the researcher and the supervisors.

### Participants

Participants in this study were individuals with overweight or obesity. Inclusion criteria were met when individuals were at least 18 years old and when the respondent’s BMI was equal to or higher than 25 kg/m^2^. Weight and length were asked to respondents, based on this information researchers calculated respondent’s BMI. Participants were recruited in cooperation with Partnerschap Overgewicht Nederland (PON) and the use social media, such as Facebook and overweight/obesity platforms. An informative text was used to inform possible respondents about the background and usefulness of the study and requirements for participation, such as the inclusion criteria. All questionnaires that were finished completely were included in the present study. The volume of the study depended on the willingness of people to participate in the study and fill in the questionnaire. This study is a non-WMO research and is therefore reviewed by the Ethics Review Committee for Health, Medicine and Life Sciences (FHML-REC) of Maastricht University. The FHML-REC has approved the protocol of the study (approval number: FHML/2020/068). All methods were carried out in accordance with relevant guidelines and regulations. An informed consent was obtained from the adult individuals with overweight and obesity who wanted to participate in this study before they filled in the questionnaire.

### Measurement and analysis

#### Cost-of-illness (COI)

The study adopted a societal perspective, which incorporates all costs, regardless who incurs them [24]. The COI followed three steps: identification, measurement and valuation.

##### Step I: identification of costs

All costs related to obesity and overweight were included. To calculate the COI different costing categories were identified. The first category is healthcare costs, defined as medical care expenditures for diagnosis, treatment, rehabilitation, and costs related to the purchase of supporting devices. The second category included the patient and family costs, i.e. transportation costs, household expenditures, clothing and informal cares of any kind [11]. The third category is other costs, such as productivity losses [11, 25].

##### Step II: measurement of costs

Overweight and obesity have a strong mental component; therefore the Treatment Inventory of Costs in Patients with psychiatric disorders (TIC-P) was used to measure costs. To keep the focus of the questionnaire on overweight and obesity and to make it complete, questions about patient and family costs were incorporated into the questionnaire. These questions elicit information about the expenditures related to the respondent’s weight, such as adapted clothing, gym subscription, diet books, parking permit, food, etc.. The TIC-P gave insight into general information, such as BMI, age, gender, and socio-economic status, and the different types of costs, such as healthcare costs, and costs in other sectors, related to obesity and overweight [26]. In short, the TIC-P related to both somatic and mental health cost items as described in step 1 “identification of costs”. Additional File 1 shows the full questionnaire in Dutch.

##### Step III: valuation of costs

The costs were gathered and calculated in Euros. The valuation of the costs was based on existing costs and cost information derived from the questionnaire. Existing costs, such as costs of medication and outpatient visits, were taken from the Dutch guidelines for costing studies in the healthcare sector [20]. In case of missing data, a conservative estimate was used. When cost data was missing the lowest cost price was used. When participants stated that they have had appointments with e.g. the dietician, but did not fill in the amount of appointments, calculations were made based on one appointment. Since the Dutch guidelines used cost prices from the year 2014, inflation was taken into account by valuation of the units. The costs were indexed to the year 2020, using rates from Statistics Netherlands. The unit costs were calculated by multiplying the unit price with the volumes of the resources used [20]. Two methods are available for calculating productivity losses, namely the Human Capital Approach (HCA) and the Friction Cost Method (FCM). In the Netherlands, the general friction period is 12 weeks [20]. Following the Dutch guidelines, the FCM method was used. Since there were no participants absent from work longer than the friction period of 12 weeks, the HCA method provide similar estimations. The calculation of the productivity loss is equal to what the employer would have paid if the individual had been working, namely the total time of absenteeism multiplied by the cost per day [27].

#### Health-related quality of life (HRQoL)

The HRQoL was measured by means of the standard Dutch version of the five-dimensional, five-level EuroQol (EQ-5D-5L). This method is recommended by the Dutch guidelines [20]. The EQ-5D-5L contains five dimensions of HRQoL, namely mobility, self-care, daily activities, pain/discomfort and depression/anxiety. Each dimension can be rated according to five scores: 1) no problems, 2) slight problems, 3) moderate problems, 4) severe problems and 5) extreme problems [28].

Disease-specific quality of life was measured using scales of the BODY-Q, which are related to overweight and obesity. A health-specific questionnaire gives more depth and insight regarding to the quality of life [20]. The BODY-Q is a Patient-Reported Outcome Measure (PROM), related to obesity and overweight. The BODY-Q is a valid, reliable and internally consistent PROM [29]. The scales that were used from the BODY-Q are all five related to overweight and obesity, namely social well-being, psychological well-being, body image, physical well-being and sexual well-being. Each statement can be rated to 4 levels ranging from totally disagree to totally agree or from never to always. It is important when answering the questions that respondents keep their body in mind.

The five dimensions of the EQ-5D-5L were summed up into a health state. Utility values can be calculated for these health states. The utility score can be valued between 0 and 1, where 0 indicated death and 1 full health. Utilities corresponding with the measured health states were derived from the Dutch tariffs [30].

The BODY-Q scales can be scored if at least half of the statements are completed; the mean is imputed when there are missing data. For each scale, a raw score was calculated; this is the sum of the levels ranging from 1 to 4. This raw score was computed and converted to a Rasch transformed score, ranging from 0 (lowest) to 100 (highest). Low Rasch scores indicate a low satisfaction with the outcome, whereas higher scores indicate a better outcome. For example, the scale psychological well-being contains 10 statements. When respondents answer all these statements with “totally agree” (score 4), the raw score will be 40, and the Rasch-score will be 100, meaning the best outcome possible.

### Subgroup analyses

Subgroup analyses were performed. These subgroups were based on gender, age, BMI, living situation, level of education and work status. The “Age” subgroup is split into 3 groups, namely 19–29, 30–49, and 50+. In the subgroup “BMI” a distinction was made between overweight (BMI ≥ 25 kg/m^2^) and obesity (BMI ≥ 30 kg/m^2^). In the subgroup “Living situation” a distinction was made between living together (meaning married or living with partner), or living alone, meaning respondents were not living with a partner (were not married nor living together), but were single (i.e. divorced, widowed, or other). Furthermore, there are 2 groups in the subgroup “Level of education”, where respondents could have a low level of education (lower vocational education, pre-vocational secondary education) and or intermediate level of education (secondary vocational education, senior secondary general education, pre-university education) – these two levels were in one group - or a high level of education (higher professional education, university education) – the second group. Last, there is a subgroup “Paid work” where respondents either have paid work or do not. The reasons for not having paid work could be that they are unemployed, retired, incapacitated, or other.

As cost data are usually skewed and not normally distributed, we had to take into account nonparametric bootstrapping (1000 replications) for all costs categories. The alpha level was set at 0.05 for all cost analyses. The Shapiro-Wilk test was used to test the normality of the HRQoL outcomes. When data was normally distributed, a parametric test (independent *t-*test) was computed. When data was not normally distributed, a nonparametric test (Mann-Whitney *U*) was computed. A value of *p* < 0.05 was considered to be a statistically significant difference. Analyses were conducted using SPSS Statistics version 24, except for bootstrapping, which was conducted with Microsoft Office Excel 2016.

## Results

Over a time period of 6 months (June – December 2020), 97 individuals filled in the questionnaire. The average age of the population was 43.31 years (SD 13.54) and 79 were female. The average BMI was 33.31 (SD 6.71). Most respondents [31] were suffering from obesity (17 respondents class 1 and 3; 18 respondents class 2); the other respondents [32] were suffering from overweight. Sixty-eight respondents indicated that they are married or living with their partner; the remaining respondents indicated that they were living alone. Forty-three respondents had a low level of education or intermediate level of education, and 54 respondents were highly educated. Most respondents (83) worked in paid employment. Respondents’ characteristics are displayed in Table 1.

**Table 1:** Participant characteristics (*N* = 97)

Participant characteristics	N (%)
Gender	
Female	79 (81.4)
Age (mean (SD))	43.32 (13.54)
19–29	23 (23.7)
30–49	34 (35.1)
50+	40 (41.2)
BMI (mean (SD))	33.31 (6.71)
Overweight (BMI ≥ 25 and ≤ 29.99 kg/m^2^)	45 (46.4)
Obesity (BMI ≥ 30 kg/m^2^)	52 (53.6)
*Obesity class 1 (BMI ≥ 30 and ≤ 34.99 kg/m*^*2*^*)*	*17 (16.5)*
*Obesity class 2 (BMI ≥ 35 and ≤ 39.99 kg/m*^*2*^*)*	*18 (17.5)*
*Obesity class 3 (BMI ≥ 40 kg/m*^*2*^*)*	*17 (16.5)*
Living situation^a^	
Living together	68 (70.1)
Highest level of education^b^	
Low and intermediate level of education	43 (44.3)
High level of education	54 (55.7)
Paid work	
Yes	83 (85.6)

^a^Living together; i.e. married or living with partner. Living alone; i.e. not living with a partner, but divorced, widowed, or other. ^b^Low and intermediate level of education: lower vocational education, pre-vocational secondary education, secondary vocational education, senior secondary general education, pre-university education. High level of education: higher professional education, university education

### Cost-of-illness (COI)

Table 2 presents an overview of the societal costs attributable to overweight and obesity, including the different costing categories. Of the respondents, 87.6% indicated that they made use of a healthcare service, this also included the use of medication or the purchase of a medical supporting device. For the whole study population the average healthcare costs per 6 months were €1453.62 (SD 3512.93). Additional File 2 provides an overview of the costing prices for the various healthcare services. Additional Files 3 and 4 show a list of all medication, both prescribed and over the counter, used by respondents.

**Table 2:** Societal costs in 2020 for people with overweight or obesity per category per 6 months

Category	Unit	Resource use		Costs per 6 months^a^ (€)
		Max	Mean (SD)	Mean (SD)
General practitioner	Consult	12	1.65 (2.34)	58.87 (83.39)
Social worker	Consult	6	0.18 (0.94)	12.32 (65.77)
Physiotherapist	Consult	32	1.71 (4.53)	61.08 (161.59)
Occupational therapist	Consult	12	0.12 (1.22)	4.42 (43.49)
Speech therapist	Consult	1	0.01 (0.10)	0.33 (3.29)
Dietitian	Consult	10	0.84 (1.81)	23.80 (51.66)
Homeopath or acupuncturist	Consult	1	0.02 (0.14)	1.65 (11.79)
Mental health institution	Consult	25	0.88 (3.64)	92.89 (385.62)
Psychologist, psychotherapist, psychiatrist – practice	Consult	25	0.43 (2.76)	44.02 (280.71)
Psychologist, psychotherapist, psychiatrist – hospital	Consult	10	0.30 (1.14)	20.69 (78.77)
Institution for addiction treatment (e.g. CAD**)	Consult	2	0.02 (0.20)	0.23 (2.23)
Company doctor	Consult	4	0.12 (0.53)	14.29 (60.70)
Hospital outpatient clinic	Consult	22	0.90 (2.79)	88.27 (274.17)
Day treatment hospital	Day	4	0.11 (0.50)	21.54 (77.65)
Other day treatment outside hospital a**	Day	12	0.15 (1.25)	69.79 (489.62)
Emergency department	Consult	10	0.19 (1.05)	60.61 (344.30)
Hospital stay	Day/night	25	0.59 (2.99)	302.53 (1536.91)
Self-help group b**	Consult	6	0.13 (0.86)	3.73 (22.12)
Medication use	Number*	9	1.46 (1.96)	74.19 (156.21)
Purchased (medical) devices	–	7	0.63 (1.22)	85.98 (326.96)
Bariatric surgery	Surgery	1	0.04 (0.20)	412.37 (1998.71)
Total healthcare costs				**1453.62 (3512.93)**
Transportation costs	–	136	15.92 (24.57)	11.18 (20.74)
Household expenditures	–			
Groceries		***	***	1031.34 (566.21)
Dining out and food delivery		***	0.81 (0.39)	350.16 (367.61)
Disabled permit	–	1	0.01 (0.10)	0.88 (8.63)
Adapted clothing	–	***	0.20 (0.40)	75.26 (177.82)
Attempt at weight loss	–	3	0.57 (0.83)	94.61 (234.29)
Informal care	Hour	1456	30.22 (158.86)	457.48 (2405.19)
Total patient and family costs				**2018.34 (2538.53)**
Productivity costs				
Absenteeism	Day	130	6.97 (23.94)	1511.93 (5885.80)
Presenteeism	Day	130	12.84 (31.58)	747.43 (2080.11)
Total other costs				**2259.37 (6141.23)**
Total societal costs				**5731.33 (8238.70)**^**b**^

All costs in Euros. *SD* Standard deviation; ^a^all prices are indexed for the year 2020. ^b^ €11,462.66 per year. *Average costs per day multiplied by days of usage. ***Resource use could be variable. **Centre for alcohol and other drug addictions. a**Dutch obesity clinic and psychiatric institution. b** Weight watchers and weight management

The average patient and family costs per 6 months were €2018.34 (SD 2538.53). Attempts at weight loss included several methods, including gym subscription, diet books, personal training, adapted diet et cetera. Thirty-four per cent of the respondents indicated that they were absent from work in the past 6 months due to sickness, and 37.1% of respondents indicated that they had physical and/or mental complaints while being present at work. This caused presenteeism. This leads to an average of total other costs of €2259.37 (SD 6141.23). The total societal costs per individual suffering from overweight or obesity in this study are €5731.33 (SD 8238.70) per 6 months, corresponding to €11,462.66 per year.

### Health-related quality of life (HRQoL)

The mean utility of the study population was 0.81 (SD 0.18). The dimension pain/discomfort is slightly affected, with 40.2% having minor problems, 16.5% having moderate problems, and a median of score 2 (minor problems). The majority of the respondents indicated that there were no or minor problems for the other dimensions, shown in Table 3.

**Table 3:** Frequencies of responded levels and utility scores for the five dimensions of the EQ-5D-5L (*N* = 97)

Dimension (Score 1–5)	Mean (SD)	Median	No problems (Score 1) N (%)	Minor problems (Score 2) N (%)	Moderate problems (Score 3) N (%)	Severe problems (Score 4) N (%)	Extreme problems (Score 5) N (%)
Mobility	1.60 (0.87)	1	59 (60.8)	23 (23.7)	10 (10.3)	5 (5.2)	0 (0.0)
Self-care	1.12 (0.42)	1	87 (89.7)	9 (9.3)	0 (0.0)	1 (1.0)	0 (0.0)
Usual activities	1.53 (0.83)	1	63 (64.9)	21 (21.6)	9 (9.3)	4 (4.1)	0 (0.0)
Pain/discomfort	1.93 (0.92)	2	36 (37.1)	39 (40.2)	16 (16.5)	5 (5.2)	1 (1.0)
Anxiety/depression	1.52 (0.83)	1	64 (66.0)	20 (20.6)	9 (9.3)	4 (4.1)	0 (0.0)
Utility score	**0.81 (0.18)**	**0.83**					

*SD* Standard variation

Table 4 shows the BODY-Q Rasch-scores for each of the HRQoL scales. The scale “Body image” scored the lowest Rasch-score (mean of 36.37), meaning a lower satisfaction with the outcome.

**Table 4:** Rasch scores of the BODY-Q

HRQoL scale (N)	Rasch-score (0–100)	Median
	Mean (SD)	
Psychological well-being (97)	60.55 (21.02)	62
Social well-being (97)	63.13 (19.63)	62
Body image (97)	36.37 (24.59)	38
Physical well-being (97)	72.93 (21.01)	71
Sexual well-being (74)^a^	58.69 (22.33)	58

^a^Non-response: 74 out of 97

*SD* Standard variation

### Subgroup analysis

#### Cost of illness (COI)

**Fig. 1:**
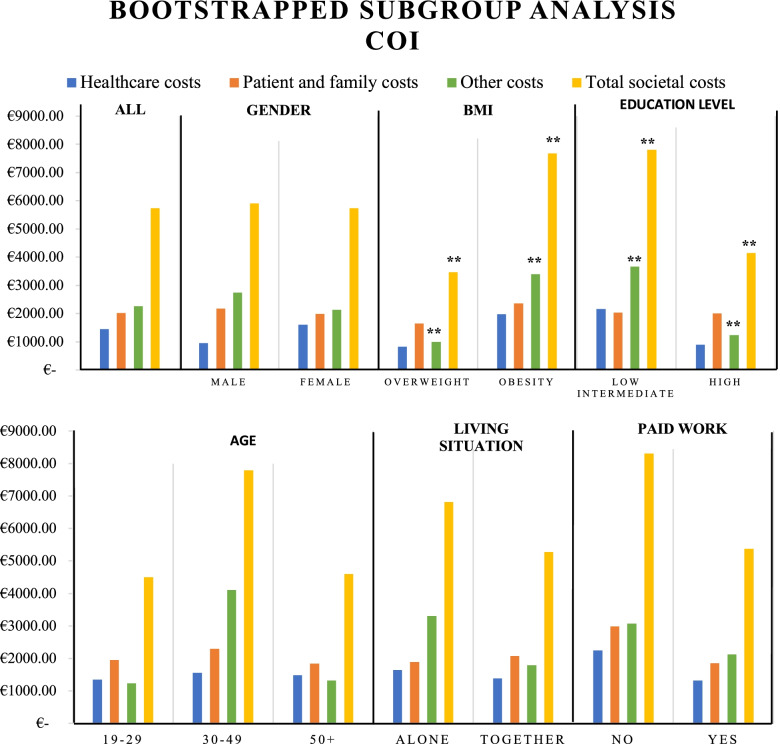
Bootstrapped subgroup analysis Cost-of-Illness (COI). *If CI includes 0, no significant difference is found. **Significant difference in costs between groups in subgroup

A subgroup analysis was performed for all relevant subgroups, based on gender, age, BMI, living situation, level of education and work status. As our costs were highly skewed, the normality assumption was violated. Therefore, bootstrapping was performed on all subgroups (Fig. 1). Additional Files 5, 6, 7, 8 show the results in more detail. Bootstrapped results showed that the other costs and total societal costs were significantly higher for respondents suffering from obesity in comparison to respondents with overweight. Furthermore, other costs and total societal costs were significantly higher for respondents with low and intermediate education in comparison with highly educated respondents.

#### Health-related quality of life (HRQoL)

**Fig. 2:**
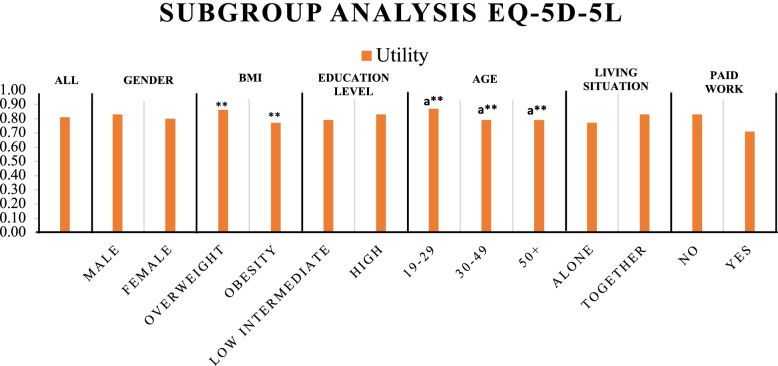
Subgroup analysis of the mean utility score derived from the five-dimensional, five-level EuroQol (EQ-5D-5L). *If *p* < 0.05, a statistically significant difference is found. **Significant difference in utility score between groups in subgroup. a**Significant difference between age group 19–29 and 30–49 and age group 19–29 and 50+

A subgroup analysis for the utility scores, derived from the EQ-5D-5L, was performed. The utility scores were not normally distributed. The Mann-Whitney *U* test was performed to test for significant differences (*p* < 0.05). Significant lower utility scores were found for respondents with obesity (0.77) in comparison to respondents with overweight (0.86). In addition, respondents in the age group of 19–29 had a significantly higher utility score (0.87) than did respondents in the other age categories (0.79). Furthermore, respondents who worked in paid employment indicated the lowest mean utility score of 0.71. The results are shown in Fig. 2. Detailed results are shown in Additional File 9.

**Fig. 3:**
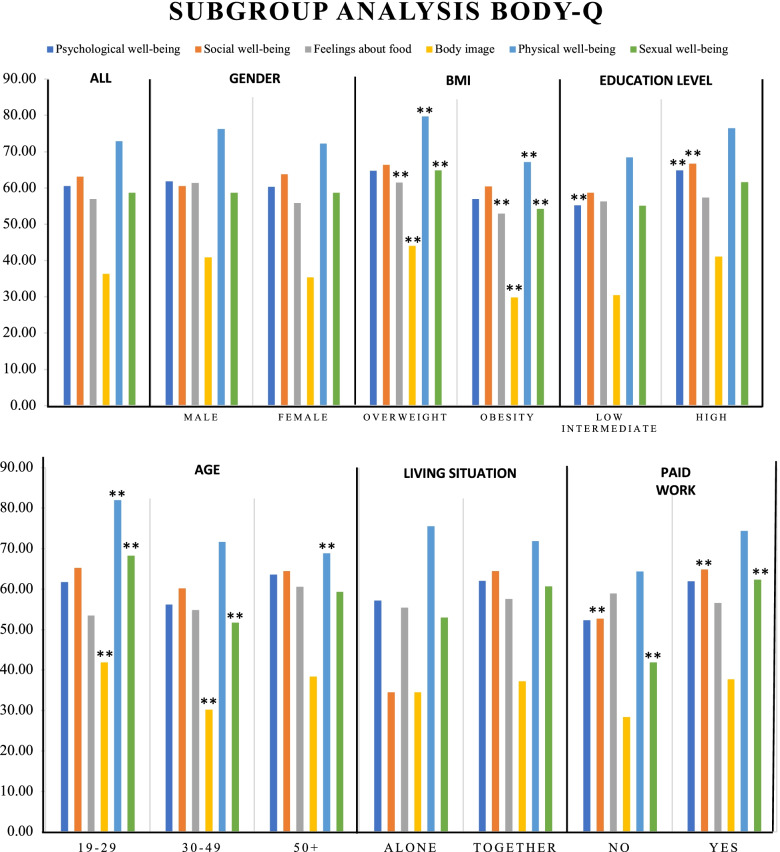
Subgroup analysis of the mean Rasch-scores derived from the BODY-Q. *If *p* < 0.05, a statistically significant difference is found. **Significant difference in Rasch-scores between groups in subgroup

Results for the subgroup analysis for the BODY-Q are shown in Fig. 3 and in more detail in Additional Files 10.1–10.5. The scales of psychological well-being, social well-being, and sexual well-being were normally distributed according to the Shapiro-Wilk test. For these scales an independent *t*-test was used to test for significant differences. The scales of body image and physical well-being were not normally distributed. For these scales the Mann-Whitney *U* test was performed to test for significant differences (*p* < 0.05). The results show that respondents with overweight have a significantly higher Rasch-score than do respondents with obesity in the scales for body image, physical well-being, and sexual well-being. In addition, there is a significant difference in the subgroup “Level of education”; respondents with a low or intermediate level of education have a significantly lower Rasch-score than respondents with higher education in the scales for psychological well-being and social well-being. Furthermore, in the scales for social well-being and sexual well-being, a significant difference is found between respondents who have paid work and those who do not, with a significantly lower Rasch-score in the latter. Last, there are significant differences in the subgroup “Age”. In the scales for body image and sexual well-being, a significantly higher Rasch-score is found for respondents aged 19–29 and 30–49. Also, in the scale for physical well-being, respondents aged 19–29 have a significantly higher Rasch-score in comparison with respondents aged 50 + .

## Discussion

This study examined the societal burden of overweight and obesity on the Dutch population in terms of COI and HRQoL. Our COI results show that the average societal costs of people with obesity and overweight are €5731.34 per person over the last 6 months, corresponding to €11,462.66 per year. Of these yearly costs, productivity losses make up the biggest share, namely €4518.7, and the healthcare costs have the lowest share, of €2907.24 per person per year, illustrating that the impact of overweight and obesity is significant beyond the healthcare sector. Our HRQoL results show a mean utility score of 0.81 for our population, derived from the EQ-5D-5L. BODY-Q results show the lowest Rasch-score of 36.37 in the scale for “Body image”. The remaining BODY-Q scales have a Rasch-score between 58.69 and 72.93.

In the Netherlands, 50% of the population has a BMI of ≥25 kg/m^2^ [2]. If we extrapolate our costs to national level, the total healthcare expenditure due to overweight and obesity is €1453.62 per capita per year. Other studies show healthcare expenditures of €290.72–€476 per capita per year in the Netherlands [13, 33]. Our study indicates that on a national level the productivity losses are €2249.37 per capita. The OECD indicates that the productivity losses are €739.19 per capita per year in the Netherlands [13]. The higher results for our study are partly due to the differences in the study design of these COI studies, i.e. top-down versus bottom-up.

Results from analysing the results from the subgroups show that in our study obesity was significantly associated with higher costs; respondents with obesity reported higher healthcare costs, patient and family costs, and significantly higher other costs and total societal costs in comparison with respondents with overweight. There are several causes for these higher costs. There are studies that indicate that obesity leads to higher healthcare costs in comparison with overweight, including costs related to diabetes and heart disease [34–36]. Analysis of the prescribed medication list (shown in Additional File 3) shows that our population also uses medication related to diabetes and heart disease. Looking at the productivity losses, studies indicate that a higher BMI is associated with more absenteeism and presenteeism, which is in line with our study. People with obesity or overweight are not only sick more often, but also longer than are people with a healthy weight [10, 37–39]. Last, persons with obesity are hampered severely in their day-to-day physical activities [15]. Our results show that respondents with obesity have higher patient and family costs than respondents with overweight. It is plausible that this result comes from the fact that respondents with obesity are more hampered in their day-to-day physical activities, and need more informal care, than do respondents with overweight.

In addition, level of education was also a significantly associated with higher costs; other costs and total societal costs were significantly higher for respondents with a low or intermediate level of education in comparison with highly educated respondents. Findings of the OECD and the Dutch Central Bureau of Statistics (CBS) indicate that a lower level of education is associated with a higher BMI [2, 40]. In our study, highly educated respondents have a slightly higher BMI (33.57) in comparison with respondents with a low or intermediate education (32.99). You could hypothesize that level of education and BMI are not related, but further research is needed to confirm this. Furthermore, our study it shows that respondents with a lower level of education are more often absent from work. Studies show that people with a lower level of education have less knowledge about health, which leads to more disabilities and higher productivity losses, resulting in higher other costs [41, 42].

The burden of obesity and overweight on the HRQoL is large if you compare the utility of our population (0.81) with the utility of the Dutch population in general (0.91–0.96), indicating that obesity and overweight carry a high burden for the respondents [30]. In our study, respondents with obesity indicate a significantly lower utility score (0.77) in comparison with respondents with overweight (0.86). According to Larsson et al. the HRQoL is negatively influenced by a higher level of obesity [43]. Furthermore, respondents in the age group of 19–29 indicate a significantly higher utility score (0.89) in comparison with the other age categories (0.79). This is in line with other studies, which show increasing problems in all EQ-5D dimensions with age [44, 45]. The HRQoL impact was highest on the dimension “Pain/discomfort”. This dimension is directly related to obesity, as people who lose weight report improved physical functioning and decreased bodily pain [15, 46]. For the disease-specific quality of life the lowest score is shown in the scale of “Body image”. Several studies show that overweight and obesity are strongly related to body dissatisfaction [32, 47, 48]. Furthermore, it was remarkable that only 74 out of 97 respondents were willing to fill in the questions about sexual well-being. According to Kolotkin et al. obesity is associated with lack of enjoyment and desire for sexual activity. In addition, obesity also leads to difficulties in sexual performance and avoidance of sexual encounters [49]. These facts could clarify the resistance to fill in these questions.

### Strengths and limitations

This study has several strengths. To the best of our knowledge, this is the first study investigating the societal burden of obesity and overweight in the Netherlands, including costs and quality of life. This study distinguishes itself from other studies by including, next to a general quality of life questionnaire, a disease-specific quality of life questionnaire, namely the BODY-Q. Furthermore this study incorporates several costs categories, such as healthcare costs, patient and family costs, and other costs (such as productivity losses). Combining this information leads to a full overview of the total burden overweight and obesity have on the society. This was a prevalence-based, bottom-up study, which means that cost units were collected on an individual level within a specific time period. In comparison to the top-down approach, the bottom-up approach has more informative power [50]. The prevalence-based approach is more useful than the incidence-based approach when the aim of the study is to draw decision-makers’ interest toward diseases whose burden is underestimated, or when the aim is to plan cost containment policies [50]. Moreover, the study is from a societal perspective, which means that all costs are taken into account. This is the most comprehensive approach and meets the principal aim of a Burden of Disease study, namely measuring the burden of the disease on society as a whole [11, 50]. Last, the EQ-5D-5L is not specific to people who are suffering from overweight and obesity, which makes it less sensitive for disease-specific effects on the quality of life. Therefore, we incorporated the BODY-Q, which makes the HRQoL in this study more specific for individuals with overweight and obesity [20].

There are some limitations in this study that need to be considered. First, we used retrospective questioning, which can lead to recall bias [51]. To minimize this bias, a time horizon of 6 months was used, because it gives the researcher the opportunity to collect relevant data and it is an acceptable time frame for participants to fill in the questionnaire with correct information. Furthermore, respondents did not always fill in the questionnaire completely, especially regarding to costs they had, which made it necessary to make an estimation of these costs. This made interpreting the actual costs harder and, therefore, it could be possible that costs are underestimated or overestimated. In addition, the sample size may preclude the use of multivariable analyses and therefore it could be possible that there might be some biases according to gender, age, socio-economic status, comorbidities, pregnancy, and/or menopause. These biases could also arise from the high portion of women relative to men in this study. Because of the small sample size and the high portion of women relative to men, it is also not possible to generalize the results to the whole Dutch population.

Last, in 2019–2020 we lived in the COVID-19 pandemic, which could have an influence on the total costs respondents incurred. In 2020 some non-essential healthcare services were not available. It could be possible that there is an underestimation of the healthcare services used, because individuals simply could not use some of these services. Furthermore, all restaurants were closed during some months in 2020–2021. This could lead to an underestimation of the patient and family costs. Regarding the other costs, it could be possible that respondents were more absent from work due to sickness, because it was not allowed to go to work with a minor cold. However, respondents filled in the questionnaire during June, July and August in 2020, which means that those questionnaires are only slightly affected regarding healthcare costs since only non-essential healthcare services were scaled down for a very short time in May 2020. Regarding patient and family costs, respondents were asked what their average monthly expenses were for dining out and food delivery. With this way of asking, we believe that most respondents did not keep the pandemic in mind and that the outcomes for patient and family costs are not or only slightly affected.

### Implications for policy, research and clinical practice

There are some implication for policy and clinical practice. First, more attention should be paid to education about the causes and influencing factors on the existence of overweight and obesity, such as a healthy lifestyle, use of medication with a side effect of increasing weight, social economic, hormonal and/or genetic factors etc. at an early age and on all levels of education [52]. The existing approach of eating less and moving more as the only solution for solving this condition is really outdated and can harm the patient even more. The impact of overweight and obesity on the HRQoL should not be neglected in treating this disease. Accordingly, it is thus important to consider the involvement of a psychologist in the treatment of overweight and obesity [31]. Last, this study again shows that overweight and obesity are complex conditions. It is important that people who suffer from overweight and obesity get effective support, such as education regarding to their disease, reimbursed access to care, a healthy workplace, and mental support in overcoming and managing this disease [53].

Since little research has been done on the burden of overweight and obesity, further research is recommended to increase knowledge on all aspects of this burden. This research should be performed when there is no pandemic. It would be interesting to make a direct comparison with the healthy population and/or other countries in future research. In addition, further exploration of the BODY-Q is needed to make a direct utility comparison with the EQ-5D-5L. Last, the use of the data gathered from this study could be important for economic evaluations in overweight and obesity.

## Conclusions

This study indicates that overweight and obesity have a considerable impact on the societal costs and HRQoL in the Netherlands. The results show that the impact of overweight and obesity go well beyond the healthcare sector, as the costs of productivity losses have the biggest share of the total societal costs of this disease. Another interesting finding of this study is that obesity leads to significant higher costs and lower HRQoL than overweight. This impact draws attention to policy making, as collective prevention and effective personalized treatment are needed to reduce this burden.

## Supplementary Information

**Additional file 1.** Questionnaire (in Dutch).

**Additional file 2.** Costing prices.

**Additional file 3.** Prescribed medication list.

**Additional file 4.** Over the counter medication list.

**Additional file 5.** Subgroup analysis of healthcare costs.

**Additional file 6.** Subgroup analysis of patient and family costs.

**Additional file 7.** Subgroup analysis of other costs.

**Additional file 8.** Subgroup analysis of total societal costs.

**Additional file 9.** Subgroup analysis utility score derived from the five-dimensional, five-level EuroQol.

**Additional file 10.** Subgroup analysis Rasch-score derived from BODY-Q, scale of psychological well-being. Subgroup analysis Rasch-score derived from BODY-Q, scale of social well-being. Subgroup analysis Rasch-score derived from BODY-Q, scale of body image. Subgroup analysis Rasch-score derived from BODY-Q, scale of physical well-being. Subgroup analysis Rasch-score derived from BODY-Q, scale of sexual well-being.

## Data Availability

The datasets generated and/or analyzed during the current study are available from the corresponding author on reasonable request.

## Abbreviations

AMA: American Medical Association
BMI: Body Mass Index
CBS: Centraal Bureau voor de Statistiek
COI: Cost of Illness
ECDC: European Centre for Disease Prevention and Control
EQ-5D-5L: Five-dimensional, Five-level EuroQol
FCM: Friction cost Method
FHML-REC: Ethics Review Committee for Health, Medicine and Life Sciences
HCA: Human Capital Approach
HRQoL: Health-related Quality of Life
OECD: Organization for Economic Cooperation and Development
PON: Partnerschap Overgewicht Nederland
TIC-P: Treatment Inventory of Costs in Patients with psychiatric disorders
