# Supplementary material for: Burden of disease study of overweight and obesity; the societal impact in terms of cost-of-illness and health-related quality of life
Source: BMC Public Health. 2022 Jan 7;22:46. doi: 10.1186/s12889-021-12449-2 (PMC8740868; doi:10.1186/s12889-021-12449-2)
Supplement: Supplementary file 1 — Additional file 1. Questionnaire (in Dutch). [file 12889_2021_12449_MOESM1_ESM.docx]

Additional File 1: Questionnaire (in Dutch)

**Instructie en informatie**

**Lees eerst de instructies voordat u verder gaat naar de vragen alstublieft!**

Overgewicht of obesitas kan verschillende beperkingen, kosten en behoeftes aan zorg met zich mee brengen. Dit onderzoek heeft als doel om de kosten, beperkingen en behoeftes in kaart te brengen die samenhangen met obesitas en overgewicht. Door middel van een vragenlijst willen we hierover zoveel mogelijk informatie verzamelen. Deze lijst bestaat uit:

- algemene vragen;
- zorg- en werk gerelateerde vragen;
- kwaliteit van leven gerelateerde vragen.

**Voor wie is deze vragenlijst?**

Deze vragenlijst is voor u. U kunt de vragenlijst per post hebben ontvangen (uw naam staat op de envelop), of u heeft de vragenlijst ontvangen via uw persoonlijk e-mailadres.

**Kunt u de lijst niet zelf invullen?**

Wanneer u de lijst niet zelf kunt invullen, kunt u eventueel om hulp vragen aan bijvoorbeeld familie of een kennis. Wanneer dit niet mogelijk is, zouden wij een afspraak kunnen inplannen om de vragenlijst telefonisch in te vullen (neem dan graag contact op met mij (Julie Hecker) door een bericht via mijn e-mail te sturen: [j.hecker@student.maastrichtuniversity.nl](mailto:j.hecker@student.maastrichtuniversity.nl))

**Waar gaat de vragenlijst over?**

De vragenlijst gaat over uw zorggebruik in de afgelopen 6 maanden en over uw werk in de afgelopen 6 maanden. Verder zullen er vragen over uw kwaliteit van leven aan bod komen. Met deze gegevens kan ik een berekening maken wat de lasten zijn voor zowel u als persoon die deze aandoening/ziekte heeft als ook voor de maatschappij. Dit kan helpen om meer aandacht te verkrijgen van verschillende instanties en overheid om dit probleem aan te pakken.

De vragenlijst begint met algemene vragen en zal daarna verder gaan met zorg- en werk- en kwaliteit van leven gerelateerde vragen.

**Hoe lang duurt het om de lijst in te vullen?**

Het duurt ongeveer 30 minuten om de lijst in te vullen.

**Hoe moet u de lijst invullen?**

- Begin bij de eerste vraag en volg de nummering.
- Kruis voor iedere vraag één hokje aan, behalve als er bij de vraag staat dat u meer dan één hokje mag aankruisen.
- Bij sommige vragen kunt u een getal of iets anders invullen op de stippellijn.
- U kunt geen foute antwoorden geven.
- U mag op elk moment stoppen met uw deelname.

**Wilt u een antwoord veranderen?**

- Streep het oude antwoord door.
- Kruis een nieuw antwoord aan.
- Zet een pijl voor het nieuwe antwoord.

Voorbeeld:

~~☒ oud antwoord~~

🡪 ☒ nieuw antwoord

**Wat gebeurt er met uw antwoorden?**

Uw antwoorden worden gebruikt voor onderzoek. Alleen de onderzoekers zien uw antwoorden.

De onderzoekers schrijven uw naam nergens op en zij zullen niemand vertellen dat u aan het onderzoek heeft meegewerkt (anonimiteit is dus gegarandeerd!).

**Vragenlijst retour sturen**

Wanneer u de lijst online invult, zal deze automatisch verzonden worden wanneer u de vragenlijst afrondt.

Wanneer u de vragenlijst op papier heeft ingevuld, kunt u de vragenlijst retour sturen in de bijgevoegde retour envelop.

**Toestemming**

Wanneer u het eens bent met onderstaande tekst, mag u uw handtekening invullen.

Bij ondertekening van dit formulier, betekent dit dat u akkoord bent met:

- Ik verklaar hierbij dat ik op een duidelijke wijze ben ingelicht over de aard, methode en doel van het onderzoek.
- Ik begrijp dat gegevens anoniem worden verwerkt en dat deze niet herleidbaar zijn tot de persoon die ze heeft ingevuld.
- Ik verklaar dat ik geheel vrijwillig aan dit onderzoek heb meegedaan en dat de uitkomsten van de vragenlijst mogen worden verwerkt in een verslag of wetenschappelijke publicatie.
- Ik begrijp dat ik op elk moment mijn deelname mag stoppen.

Handtekening…………………………………………………………………………………..

Wanneer u vragen heeft of wanneer er onduidelijkheden zijn, dan kunt u contact opnemen met mij (Julie Hecker) via mijn e-mailadres: [j.hecker@student.maastrichtuniversity.nl](mailto:j.hecker@student.maastrichtuniversity.nl).

**Bij voorbaat dank voor uw medewerking!**

**Deel 1: Algemene vragen**

**Vraag 1. Op welke datum vult u deze vragenlijst in?**

*Voorbeeld: 5 mei 2020 = 05 05 2020*

Dag Maand Jaar

**. . . . . . . .**

**vraag 2. Wat is uw geboortedatum?**

*Voorbeeld: 5 maart 1970 = 05 03 1970*

Dag Maand Jaar

**. . . . . . . .**

**Vraag 3. Wat is uw geslacht?**

Man

Vrouw

**Vraag 4. Wat is op dit moment uw burgerlijke staat**

Ik ben getrouwd of ik woon samen met mijn partner

Ik ben niet getrouwd en woon ook niet samen

Ik ben gescheiden

Ik ben weduwe/weduwnaar

Overig, namelijk………………………………………………….

**Vraag 5.** **Wat is de hoogste opleiding die u heeft afgemaakt?**

Geen school of opleiding afgemaakt

Lager onderwijs (basisschool, speciaal onderwijs)

Lager beroepsonderwijs (huishoudschool, vbo, lbo, leao, lhno)

Middelbaar algemeen onderwijs (mavo, mulo, ivo of vmbo)

Middelbaar beroepsonderwijs (mbo, mts, meao, mhno, inas of intas)

Voortgezet algemeen onderwijs (hbs, mms, havo, vwo, atheneum of gymnasium)

Hoger beroepsonderwijs (hbo, hts, heao, hhno)

Universiteit

Ik heb een andere opleiding afgemaakt, namelijk ……………………………

…………………………………………………………………………………………………………….

**Vraag 6. Wat doet u in het dagelijks leven?** Kruis aan wat u de meeste tijd doet.

Ik zit op school

Ik werk in loondienst

Ik ben zelfstandig ondernemer

Ik ben huisvrouw, huisman

Ik ben werkloos

Ik ben arbeidsongeschikt, voor ….. %

Ik ben met pensioen of prepensioen

Ik doe iets anders, namelijk ………………………………………………………………

…………………………………………………………………………………………………………….

**Vraag 7. Wat is uw lengte en gewicht?**

*Voorbeeld: 1 meter 60 = 160 cm.*

……….. cm ……….. kg

**Vraag 8. Welke eventuele lichamelijke en psychische klachten heeft u?** *Kruis aan welke klachten u nu ervaart. Kruis ook aan welke klachten u nog meer heeft gehad in de afgelopen 6 maanden. U kunt dus meer dan 1 hokje aankruisen.*

**Hart en bloedvaten**

Gevolgen van hartinfarct

Ernstige hartproblemen

Hoge bloeddruk

Gevolgen van beroerte

Anders, namelijk ……………………………………………………………………………………………

Geen van bovenstaande

**Maag en darmen**

Maagzweer

Zweer in de 12-vingerige darm

Ernstige darmproblemen die langer dan 3 maanden duurde

Anders, namelijk ……………………………………………………………………………………………

Geen van bovenstaande

**Galblaas, lever en nieren**

Galstenen of galblaasontsteking

Leverziekte of levercirrose

Nierstenen

Ernstige nierziekte

Anders, namelijk ……………………………………………………………………………………………

Geen van bovenstaande

**Blaas en baarmoeder**

Blaasontsteking die langer dan 3 maanden duurde of die steeds terugkomt

Verzakking

Anders, namelijk ……………………………………………………………………………………………

Geen van bovenstaande

**Longen en hoofdholten**

Astma, blijvende bronchitis of CARA

Ontsteking in de neusbijholte, voorhoofdsholte of kaakholten

Anders, namelijk ……………………………………………………………………………………………

Geen van bovenstaande

**Rug en gewrichten**

Hernia

Andere rugpijn die langer dan 3 maanden duurde

Artrose aan uw knieën, heupen of handen. Artrose is slijtage aan uw gewrichten.

Reuma aan uw handen of voeten. Bij reuma zijn uw gewrichten ontstoken.

Andere reuma die langer dan 3 maanden duurde

Anders, namelijk ……………………………………………………………………………………………

Geen van bovenstaande

**Zenuwstelsel**

Epilepsie

Andere ziekte van het zenuwstelsel. Bijvoorbeeld de ziekte van Parkinson of multiple sclerose

Duizeligheid waardoor u regelmatig valt

Migraine

Anders, namelijk ……………………………………………………………………………………………

Geen van bovenstaande

**Andere ziektes**

Kanker

Suikerziekte

Problemen met de schildklier

Eczeem of huidziekte die langer dan 3 maanden duurde.

Anders, namelijk ……………………………………………………………………………………………

Geen van bovenstaande

**Andere lichamelijke klachten**

Verwondingen, *voorbeeld: gebroken pols, gekneusde rib, gescheurde enkelband.*

Anders, namelijk ……………………………………………………………………………………………

Geen van bovenstaande

**Psychische klachten**

Overspannen zijn, burn-out

Depressie

Anders, namelijk ……………………………………………………………………………………………

Geen van bovenstaande

**Deel 2:** **Vragen over uw zorggebruik**

De volgende vragen zijn bedoeld om in kaart te brengen met welke zorg- en/of hulpverleners u in de afgelopen 6 maanden contact heeft gehad. Het gaat hierbij om afspraken voor **uzelf.**

**Welke afspraken tellen mee?**

- Controles
- Afspraken omdat u een lichamelijke of psychische klacht had
- Afspraken waarbij de dokter bij u thuis kwam
- Telefonische afspraken
- Telefoontjes met de receptenlijn.

**Welke afspraken tellen niet mee?**

- Telefonische afspraken om een afspraak te maken
- Contacten om een afspraak te maken voor een ander.

Wanneer u een antwoord niet precies weet, mag u een **schatting** geven.

**Vraag 1.** **Hoeveel afspraken had u in de afgelopen 6 maanden met uw huisarts?** *Spreekuurbezoek, bezoek op afspraak, huisbezoek en telefonische consulten bij elkaar optellen.*

Geen afspraken

**…….** afspraken

**Vraag 2.** **Hoeveel afspraken/contact had u in de afgelopen 6 maanden met een maatschappelijk werk(st)er?**

Geen afspraken

**…….** afspraken

**Vraag 3.** **Hoeveel afspraken had u in de afgelopen 6 maanden met een fysiotherapeut, caesartherapeut, therapeut mensendieck of een manueel therapeut?** *Tel alle afspraken bij elkaar op.*

Geen afspraken

**…….** afspraken

**Vraag 4****. Hoeveel afspraken had u in de afgelopen 6 maanden met een ergotherapeut?**

Geen afspraken

**…….** afspraken

**Vraag 5.** **Hoeveel afspraken had u in de afgelopen 6 maanden met een logopedist?**

Geen afspraken

**…….** Afspraken

**Vraag 6.** **Hoeveel afspraken had u in de afgelopen 6 maanden met een diëtist?**

Geen afspraken

**…….** afspraken

**Vraag 7.** **Hoeveel afspraken had u in de afgelopen 6 maanden met een homeopaat of met een acupuncturist?** *Tel alle afspraken bij elkaar op.*

Geen afspraken

**…….** Afspraken, totale kosten hiervoor € ………………….

**Vraag 8.** **Hoeveel afspraken had u in de afgelopen 6 maanden bij het RIAGG of bij een andere instelling van GGZ?**

Geen afspraken

**…….** afspraken

**Vraag 9.** **Hoeveel afspraken had u in de afgelopen 6 maanden met een psycholoog, een psychotherapeut of psychiater met een EIGEN praktijk?** *Tel alle afspraken bij elkaar op.*

Geen afspraken

**…….** afspraken

**Vraag 10.** **Hoeveel afspraken had u in de afgelopen 6 maanden met een psycholoog, psychotherapeut of psychiater in het ZIEKENHUIS?** *Het gaat om afspraken bij de polikliniek. Tel alle afspraken bij elkaar op.*

Geen afspraken

**…….** afspraken

**Vraag 11.** **Hoeveel afspraken had u in de afgelopen 6 maanden bij een Consultatiebureau voor Alcohol en Drugs (CAD) of bij een andere instelling voor verslavingszorg?**

Geen afspraken

**…….** afspraken

**Vraag 12.** **Hoe vaak bent u in de afgelopen 6 maanden bij een zelfhulpgroep geweest?** *Voorbeeld: Ladyline, WeightWatchers (WW), A.A.-groep etc.*

Geen afspraken

Ja, namelijk

| Soort zelfhulpgroep | Aantal afspraken in afgelopen 6 maanden | Geschatte kosten |
| --- | --- | --- |
|  |  | € |
|  |  | € |
|  |  | € |

**Vraag 13.** **Hoeveel afspraken had u in de afgelopen 6 maanden bij een bedrijfsarts?**

Geen afspraken

**…….** afspraken

**Vraag 14.** **Heeft u in de afgelopen 6 maanden hulp van thuiszorg gehad?**

Nee (u mag meteen verder naar vraag 18)

Ja (beantwoord vraag 15 t/m 17)

**Vraag 15.** **Wat voor hulp van de thuiszorg heeft u gehad in de afgelopen 6 maanden?** *U kunt meer dan één hokje aankruisen.*

Huishoudelijke hulp

*Voorbeeld: poetsen, bed opmaken, boodschappen doen.*

Verzorging van uzelf

*Voorbeeld: hulp bij douchen en/of aankleden.*

Verpleging

*Voorbeeld: wond verzorging, bloeddruk meten, medicijnen geven.*

Anders, namelijk………………………………………………………………………………………………

**Vraag 16.** **Hoeveel weken heeft u deze thuiszorg gehad?** *Tel alle weken in de afgelopen 6 maanden bij elkaar op.*

Huishoudelijke hulp: …. weken in de afgelopen 6 maanden

Verzorging voor uzelf: …. weken in de afgelopen 6 maanden

Verpleging: …. weken in de afgelopen 6 maanden

Anders: …. Weken in de afgelopen 6 maanden

**Vraag 17.** **Hoeveel uur thuiszorg kreeg u in deze weken gemiddeld?**

Huishoudelijke hulp: gemiddeld …. uur in de week

Verzorging voor uzelf: gemiddeld …. uur in de week

Verpleging: gemiddeld …. uur in de week

Anders: gemiddeld …. Uur in de week

**Vraag 18.** **Heeft u in de afgelopen 6 maanden medicijnen gebruikt?** *Medicijnen tijdens ziekenhuisopname NIET meerekenen, evenmin als de anticonceptie pil.*

Nee

Ja

Heeft u “Ja” gekozen? Vul dan vraag 19 in. Heeft u “Nee” gekozen, ga dan naar vraag 20.

**Vraag 19.** **Welke medicijnen heeft u in de afgelopen 6 maanden gebruikt?** *Met medicijnen bedoelen we alle medicijnen die u op recept gekregen heeft en geneesmiddelen die u hebt gekocht bij de apotheek of drogist. Pakt u zo mogelijk de verpakking van de medicatie erbij, daarop staat hoeveel u per keer moest innemen en hoe vaak u dit moest doen.* ***Heeft u meer of minder gebruikt? Vul dan in hoeveel u echt gebruik heeft.***

| Medicijn (naam) | Dosis per inname | Aantal keren per dag | Aantal dagen afgelopen 6 maanden |
| --- | --- | --- | --- |
| *Voorbeeld:*  *Metoprolol* | *100 mg* | *1 keer* | *90 dagen* |
|  |  |  |  |
|  |  |  |  |
|  |  |  |  |
|  |  |  |  |
|  |  |  |  |
|  |  |  |  |
|  |  |  |  |
|  |  |  |  |
|  |  |  |  |
|  |  |  |  |

**Vraag 20.** **Had u in de afgelopen 6 maanden een afspraak bij de polikliniek van het ziekenhuis?** *Het gaat om afspraken voor uzelf met een dokter, bijvoorbeeld een reumatoloog, cardioloog, neuroloog etc.*

***Let op:*** *afspraken met psycholoog, psychotherapeut of psychiater tellen* ***niet*** *mee!*

Nee

Ja

Heeft u “Ja” gekozen? Vul dan vraag 21 in. Heeft u “Nee” gekozen, ga dan verder naar vraag 22.

**Vraag 21.** **Bij welke soort dokter bent u in de afgelopen 6 maanden in het ziekenhuis geweest, en hoe vaak?**

| Soort dokter in het ziekenhuis | Hoe vaak bent u bij deze dokter geweest in de afgelopen 6 maanden |
| --- | --- |
| *Voorbeeld: cardioloog* | *Voorbeeld: 2 keer* |
|  |  |
|  |  |
|  |  |
|  |  |
|  |  |
|  |  |

**Vraag 22.** **Bent u in de afgelopen 6 maanden overdag in het ziekenhuis geweest voor behandeling?** *U bleef dus niet slapen. U kwam bijvoorbeeld voor nierdialyse, chemokuur, bloedtransfusie etc.*

Nee

Ja

Heeft u “Ja” gekozen? Beantwoord dan vraag 23. Heeft u “Nee” gekozen, ga dan verder naar vraag 24.

**Vraag 23.** **Voor welke soort behandeling was dit en hoeveel keer moest u deze ondergaan in de afgelopen 6 maanden?**

| Soort behandeling | Hoe vaak bent u voor deze behandeling geweest in de afgelopen 6 maanden |
| --- | --- |
| *Voorbeeld: nierdialyse* | *100 keer* |
|  |  |
|  |  |
|  |  |

**Vraag 24.** **Bent u in de afgelopen 6 maanden ergens anders geweest voor behandeling overdag?** *U bleef dus niet slapen. U ging bijvoorbeeld naar de dagopvang van een woon-/zorgcentrum of een psychiatrische instelling. Of naar de dagbehandeling van een revalidatiecentrum.*

Nee

Ja

Heeft u “Ja” gekozen? Beantwoord dan vraag 25 en 26. Heeft u “Nee” gekozen, ga dan verder naar vraag 27.

**Vraag 25.** **Wat voor een instelling was dit?** *Kruis het goede antwoord aan. U kunt meer dan één hokje aankruisen.*

Woon-/zorgcentrum

Revalidatiecentrum

Psychiatrische instelling

Anders, namelijk …………………………………………………………………………….

…………………………………………………………………………………………………………..

**Vraag 26.** **Hoe vaak moest u hier in de afgelopen 6 maanden naartoe?** *Heeft u bij vraag 25 meer dan één hokje aangekruist? Vul dan hieronder voor iedere instelling in hoe vaak u er bent geweest.*

Naar het woon-/zorgcentrum: …. keer in de afgelopen 6 maanden

Naar het revalidatiecentrum: …. keer in de afgelopen 6 maanden

Naar psychiatrische instelling: …. keer in de afgelopen 6 maanden

Naar de andere instelling: …. keer in de afgelopen 6 maanden

**Vraag 27.** **Hoe vaak bent u in de afgelopen 6 maanden op de spoedeisende eerste hulp (EHBO) van een ziekenhuis geweest?**

Geen enkele keer

…….. keer

**Vraag 28.** **Heeft u in de afgelopen 6 maanden in het ziekenhuis gelegen?** *U moest dus blijven slapen. Bijvoorbeeld omdat u geopereerd was en niet meteen naar huis kon.*

Nee

Ja

Heeft u “Ja” gekozen? Beantwoord dan vraag 29 en 30. Heeft u “Nee” gekozen, ga dan verder met vraag 31.

**Vraag 29.** **Hoe vaak heeft u in de afgelopen 6 maanden in het ziekenhuis gelegen?**

….. keer in de afgelopen 6 maanden.

**Vraag 30.** **Hoe lang heeft u in het ziekenhuis gelegen?** *Heeft u meer dan één keer in het ziekenhuis gelegen de afgelopen 6 maanden? Tel dan alle dagen bij elkaar op.*

….. dagen in de afgelopen 6 maanden.

**Vraag 31.** **Moest u in de afgelopen 6 maanden ergens anders blijven slapen voor uw gezondheid?** *Bijvoorbeeld in een woon-/zorgcentrum, psychiatrische instelling of revalidatiecentrum.*

Nee

Ja

Heeft u “Ja” gekozen? Beantwoord dan vraag 32 en 33. Heeft u “Nee” gekozen, ga dan verder naar deel 3: vragen over uw werk.

**Vraag 32.** **Wat voor een instelling was dit?** *U kunt meer dan één hokje aankruisen.*

Woon-/zorgcentrum

Revalidatiecentrum

Psychiatrische instelling

Anders, namelijk …………………………………………………………………………….

…………………………………………………………………………………………………………..

**Vraag 33.** **Hoe lang bent u in deze instelling geweest?** *Heeft u bij vraag 32 meer dan één hokje aangekruist? Vul dan hieronder voor iedere instelling in hoe lang u er bent geweest.*

*Bent u ergens meer dan één keer geweest de afgelopen 6 maanden? Tel dan alle dagen bij elkaar op.*

In het woon-/zorgcentrum: …. dagen in de afgelopen 6 maanden

In het revalidatiecentrum: …. dagen in de afgelopen 6 maanden

In psychiatrische instelling: …. dagen in de afgelopen 6 maanden

In de andere instelling: …. dagen in de afgelopen 6 maanden

**Deel 3: Vragen over uw werk**

De volgende vragen gaan over uw werk in de periode van de afgelopen 6 maanden.

Eerst krijgt u vragen over uw betaalde werk. Heeft u geen betaald werk? Ga dan verder naar vraag 11. Lees eerst de toelichting boven vraag 11.

**Vraag 1. Wat is uw beroep?**

**……………………………………………………………………………………………………………**

**Vraag 2.** **Hoeveel uur per week werkt u?** *Tel alleen de uren waarvoor u betaald wordt.*

**…………** uren

**Vraag 3.** **Hoeveel dagen in de week werkt u?**

**…………** dagen

**Vraag 4.** **Wat is uw eigen netto inkomen uit betaald werk?**

**€……….** per maand

Weet ik niet of wil ik niet zeggen.

**Vraag 5.** **Bent u in de afgelopen 6 maanden afwezig geweest van uw werk omdat u ziek was?**

Nee

Ja, ik ben …… dagen afwezig geweest *(tel alleen de werkdagen in de afgelopen 6 maanden).*

Heeft u “Ja” gekozen? Beantwoord dan vraag 6. Heeft u “Nee” gekozen, ga dan verder naar vraag 8.

**Vraag 6.** **Was u langer dan de gehele periode van 6 maanden afwezig van uw werk omdat u ziek was?** *Het gaat om een aaneengesloten periode van werkverzuim.*

Nee

Ja

Heeft u “Ja” gekozen? Beantwoord dan vraag 7. Heeft u “Nee” gekozen, ga dan verder naar vraag 8.

**Vraag 7. Wanneer heeft u zich ziek gemeld?**

*Voorbeeld: 5 maart 2019 = 05 03 2019*

Dag Maand Jaar

**. . . . . . . .**

Ga verder met vraag 11. Lees eerst de toelichting boven vraag 11.

**Vraag 8.** **Waren er in de afgelopen 6 maanden dagen waarop u wel gewerkt heeft, maar tijdens uw werk last had van lichamelijke en/of psychische klachten?**

Nee

Ja

Heeft u “Ja” gekozen? Beantwoord dan vraag 9 en 10. Heeft u “Nee” gekozen, ga dan verder naar vraag 11. Lees eerst de toelichting boven vraag 11.

**Vraag 9.** **Op hoeveel werkdagen had u tijdens uw werk last van uw lichamelijke en/of psychische klachten?** *Tel alleen de werkdagen in de afgelopen 6 maanden.*

**…………** werkdagen

**Vraag 10.** **Op de dagen dat u lichamelijke en/of psychische klachten had, kon u misschien niet zoveel werk doen als normaal. Hoeveel werk kon u op deze dagen gemiddeld doen?** *Kijk naar de cijfers hieronder. Een 10 betekent dat u op deze dagen net zoveel kon doen als normaal. Een 0 betekent dat u op deze dagen niets kon doen. zet een kruis in het vakje onder het goede cijfer.*

| 0 | 1 | 2 | 3 | 4 | 5 | 6 | 7 | 8 | 9 | 10 |
| --- | --- | --- | --- | --- | --- | --- | --- | --- | --- | --- |
|  |  |  |  |  |  |  |  |  |  |  |

**Toelichting**

Ook bij onbetaald werk kunt u last hebben van lichamelijke en/of psychische klachten. Soms kunt u daardoor minder doen. U kunt bijvoorbeeld niet goed voor de kinderen zorgen, boodschappen doen, in de tuin werken, schoonmaken of vrijwilligerswerk doen. De volgende vragen gaan hierover.

**Vraag 11.** **Waren er dagen waarop u minder onbetaald werk kon doen door uw lichamelijke en/of psychische klachten?** *Het gaat om de dagen in de afgelopen 6 maanden.*

Nee

Ja

Heeft u “Ja” gekozen? Beantwoord dan vraag 12 en 13. Heeft u “Nee” gekozen, ga dan verder naar deel 4 van de vragenlijst.

**Vraag 12.** **Op hoeveel dagen was dit zo?** *Tel alleen de dagen in de afgelopen 6 maanden.*

**…………** dagen

**Vraag 13.** **Heeft iemand, bijvoorbeeld uw partner, familielid, bekende, of betaalde hulp u deze dagen geholpen. En al het onbetaalde werk wat u niet kon doen, voor u had gedaan. Hoeveel uur was die persoon hier op deze dagen dan gemiddeld mee bezig geweest?**

Gemiddeld ……….. uur op deze dagen.

**Deel 4: Uitgaven in verband met uw gewicht.**

**Vraag 1.** **Heeft u in de afgelopen 6 maanden een van deze hulpmiddelen moeten aanschaffen in verband met uw gewicht?** **Zo “Ja” wat waren de geschatte kosten?**

| Hulpmiddel | Ja | Nee | Geschatte kosten |
| --- | --- | --- | --- |
| Aangepast toilet |  |  | € |
| Aangepaste douche (bijv. veiligheidshandgrepen) |  |  | € |
| Verbreedde stoel |  |  | € |
| Verbreed bed |  |  | € |
| Apneu respirator |  |  | € |
| Astma inhaler |  |  | € |
| Traplift |  |  | € |
| (aangepaste) Rolstoel |  |  | € |
| (aangepaste) Rollator |  |  | € |
| (aangepaste) Scootmobiel |  |  | € |
| Extra grote kleerhangers |  |  | € |
| Een hulpmiddel om sokken aan te trekken |  |  | € |
| Talkpoeder |  |  | € |
| Incontinentie maandverband |  |  | € |
| Steunzolen |  |  | € |
| Speciale (brede) sokken |  |  | € |
| Ondersteunende schoenen |  |  | € |
| Vitaminesupplementen |  |  | € |
| Anders, namelijk………………………………………………. |  |  | € |

**Vraag 2.** **Heeft u door uw gewicht in de afgelopen 6 maanden extra kosten gehad bij het aanschaffen van kleding? Indien “Ja”, wat waren de geschatte kosten?**

❏ Nee

❏ Ja, namelijk €…………….

**Vraag 3.** **Heeft u de afgelopen 6 maanden pogingen ondernomen om af te vallen? Indien “Ja”, hoeveel en wat voor een pogingen waren dit?** *Denk aan dieet, kopen van dieetboeken, kopen van shakes, deelnemen aan afvalclubje of abonnement sportschool. U mag een schatting geven.*

❏ Nee (*Gaat u verder met vraag 5*)

❏ Ja, namelijk………….afvalpogingen (tel alle pogingen die u in onderstaande kolom invult bij elkaar op)

- 1. **Kunt u aangeven door middel van welke producten/services u heeft geprobeerd af te vallen en welke kosten hieraan verbonden zitten?** *(Bijv. door het aanschaffen van boeken, shakes, een bepaald dieet, sportschool abonnement, afvalclub etc.)*

| Hoe? *Dieet, aanschaffen boeken, shakes, afvalclubje, sportschool abonnement* | Geschatte kosten afgelopen 6 maanden |
| --- | --- |
| *Voorbeeld: sportschoolabonnement* | **€35 x 6 maanden = €210** |
|  | **€** |
|  | **€** |
|  | **€** |
|  | **€** |

**Vraag 4.** **Hebt u voor uw auto een vergunning om op invalide parkeerplaatsen te parkeren in verband met uw gewicht? Hoeveel kost deze vergunning?**

❏ Nee

❏ Ja, voor €…………………..

**Vraag 5.** **Welk bedrag besteedt u maandelijks aan boodschappen? Geef hierbij ook aan uit hoeveel gezinsleden uw gezin bestaat waar u de boodschappen voor doet.***Tip: U kunt eenvoudig een indicatie van uw maandelijkse uitgaven krijgen door uw bankafschriften erbij te pakken.*

€…………….

Aantal gezinsleden: ……….

**Vraag 6.** **Welk bedrag besteedt u, voor u alleen, maandelijks aan eten buiten de deur** *(bijv. uit eten, afhalen, drive-in, fastfood ketens)?*

€…………….

**Vraag 7.** **Welk bedrag besteedt u, voor u alleen, maandelijks aan eten thuis laten bezorgen (*bijv. thuisbezorgd.nl of pizza*)?**

€…………….

**Deel 5: Vragen over uw kwaliteit van leven**

**Zet bij iedere groep in de lijst hieronder een kruisje in het hokje achter de zin die het beste past bij uw eigen gezondheid vandaag.**

**MOBILITEIT**

Ik heb geen problemen met lopen ❑

Ik heb een beetje problemen met lopen ❑

Ik heb matige problemen met lopen ❑

Ik heb ernstige problemen met lopen ❑

Ik ben niet in staat om te lopen ❑

**ZELFZORG**

Ik heb geen problemen met mijzelf wassen of aankleden ❑

Ik heb een beetje problemen met mijzelf wassen of aankleden ❑

Ik heb matige problemen met mijzelf wassen of aankleden ❑

Ik heb ernstige problemen met mijzelf wassen of aankleden ❑

Ik ben niet in staat mijzelf te wassen of aan te kleden ❑

**DAGELIJKSE ACTIVITEITEN *(bijv. werk, studie, huishouden, gezins- en vrijetijdsactiviteiten)***

Ik heb geen problemen met mijn dagelijkse activiteiten ❑

Ik heb een beetje problemen met mijn dagelijkse activiteiten ❑

Ik heb matige problemen met mijn dagelijkse activiteiten ❑

Ik heb ernstige problemen met mijn dagelijkse activiteiten ❑

Ik ben niet in staat mijn dagelijkse activiteiten uit te voeren ❑

**PIJN/ONGEMAK**

Ik heb geen pijn of ongemak ❑

Ik heb een beetje pijn of ongemak ❑

Ik heb matige pijn of ongemak ❑

Ik heb ernstige pijn of ongemak ❑

Ik heb extreme pijn of ongemak ❑

**ANGST/SOMBERHEID**

Ik ben niet angstig of somber ❑

Ik ben een beetje angstig of somber ❑

Ik ben matig angstig of somber ❑

Ik ben erg angstig of somber ❑

Ik ben extreem angstig of somber ❑

- We willen weten hoe goed of slecht uw gezondheid VANDAAG is.
- Deze meetschaal (te vergelijken met een thermometer) loopt van 0 tot 100.
- 100 staat voor de beste gezondheid die u zich kunt voorstellen.
  0 staat voor de slechtste gezondheid die u zich kunt voorstellen.
- Plaats een kruisje op de meetschaal om aan te geven hoe goed of hoe slecht uw gezondheid VANDAAG is.
- Noteer het getal waarbij u de X heeft geplaatst in onderstaand vakje.

uw gezondheid VANDAAG = ❑

10

0

20

30

40

50

60

80

70

90

100

5

15

25

35

45

55

75

65

85

95

**De beste gezondheid die u zich kunt voorstellen**

**De slechtste gezondheid die u zich kunt voorstellen**

**Deze stellingen gaan over de kwaliteit van leven in relatie tot uw gewicht. Zet een rondje om het door u gekozen antwoord.**

1. **Uw psychisch welbevinden:**

**Als u aan uw lichaam denkt, in de afgelopen week, in hoeverre bent u het oneens of eens met iedere stelling:**

|  | **Helemaal oneens** | **Een beetje oneens** | **Een beetje eens** | **Helemaal eens** |
| --- | --- | --- | --- | --- |
| 1. Ik geloof in mijzelf. | 1 | 2 | 3 | 4 |
| 2. Ik ben trots op mijzelf. | 1 | 2 | 3 | 4 |
| 3. Ik voel me gelukkig. | 1 | 2 | 3 | 4 |
| 4. Ik vind mijzelf leuk. | 1 | 2 | 3 | 4 |
| 5. Ik ben emotioneel sterk. | 1 | 2 | 3 | 4 |
| 6. Ik heb het gevoel dat ik mijn leven in de hand heb. | 1 | 2 | 3 | 4 |
| 7. Ik voel me zelfverzekerd. | 1 | 2 | 3 | 4 |
| 8. Ik accepteer mijzelf | 1 | 2 | 3 | 4 |
| 9. Ik voel me op mijn gemak met mijzelf. | 1 | 2 | 3 | 4 |
| 10. Ik vind mijzelf geweldig. | 1 | 2 | 3 | 4 |

1. **Sociaal welzijn.**

**Als u aan uw lichaam denkt, in de afgelopen week, in hoeverre bent u het oneens of eens met iedere stelling:**

|  | **Helemaal oneens** | **Een beetje oneens** | **Een beetje eens** | **Helemaal eens** |
| --- | --- | --- | --- | --- |
| 1. Ik voel me op mijn gemak op sociale bijeenkomsten met mensen die ik ken. | 1 | 2 | 3 | 4 |
| 2. Mensen luisteren naar wat ik te zeggen heb. | 1 | 2 | 3 | 4 |
| 3. Ik voel me geaccepteerd door andere mensen. | 1 | 2 | 3 | 4 |
| 4. Ik voel me erbij horen in sociale situaties. | 1 | 2 | 3 | 4 |
| 5. Ik maak een goede eerste indruk. | 1 | 2 | 3 | 4 |
| 6. Ik neem deel aan het leven in plaats van achterover te leunen. | 1 | 2 | 3 | 4 |
| 7. Ik maak gemakkelijk nieuwe vrienden. | 1 | 2 | 3 | 4 |
| 8. Ik ben zelfverzekerd in een groep (bijvoorbeeld vergaderingen). | 1 | 2 | 3 | 4 |
| 9. ik ben ontspannen tussen mensen die ik niet goed ken. | 1 | 2 | 3 | 4 |
| 10. Ik voel me zelfverzekerd wanneer ik een ruimte vol mensen inga die ik niet ken. | 1 | 2 | 3 | 4 |

1. **Het beeld dat u van uw lichaam heeft.**

**Als u aan uw lichaam denkt, in de afgelopen week, in hoeverre bent u het oneens of eens met iedere stelling:**

|  | **Helemaal oneens** | **Een beetje oneens** | **Een beetje eens** | **Helemaal eens** |
| --- | --- | --- | --- | --- |
| 1. Ik denk positief over mijn lichaam. | 1 | 2 | 3 | 4 |
| 2. Mijn lichaam is niet perfect, maar ik hou ervan. | 1 | 2 | 3 | 4 |
| 3. Ik ben blij met mijn lichaam. | 1 | 2 | 3 | 4 |
| 4. Ik ben trots op mijn lichaam. | 1 | 2 | 3 | 4 |
| 5. Ik vind mijn lichaam aantrekkelijk. | 1 | 2 | 3 | 4 |
| 6. Ik voel me goed over mijn lichaam als ik naakt ben. | 1 | 2 | 3 | 4 |
| 7. Ik heb het lichaam dat ik wil. | 1 | 2 | 3 | 4 |

1. **Lichamelijke activiteiten.**

**Als u aan uw lichaam denkt, in de afgelopen week, hoe vaak heeft u problemen gehad met:**

|  | **Altijd** | **Vaak** | **Soms** | **Nooit** |
| --- | --- | --- | --- | --- |
| 1. Opstaan uit bed? | 1 | 2 | 3 | 4 |
| 2. Buigen van de ene kant naar de andere? | 1 | 2 | 3 | 4 |
| 3. Wandelen en rondlopen? | 1 | 2 | 3 | 4 |
| 4. Naar voren buigen (bijvoorbeeld om uw schoenen vast te maken)? | 1 | 2 | 3 | 4 |
| 5. Matige lichamelijke inspanning (bijvoorbeeld een stevige wandeling maken)? | 1 | 2 | 3 | 4 |
| 6. De trap op- en aflopen? | 1 | 2 | 3 | 4 |
| 7. Langdurig staan? | 1 | 2 | 3 | 4 |

1. **Seksueel welzijn.**

|  | **Ja** | **Nee** |
| --- | --- | --- |
| 1. Wilt u deze vragen beantwoorden? | 1 | 2 |

**Als u aan uw lichaam denkt, in hoeverre bent u het oneens of eens met iedere stelling?**

|  | **Helemaal oneens** | **Een beetje oneens** | **Een beetje eens** | **Helemaal eens** |
| --- | --- | --- | --- | --- |
| 1. Ik vind seks bevredigend | 1 | 2 | 3 | 4 |
| 2. Ik ben op mijn gemak als ik me voor mijn partner uitkleed. | 1 | 2 | 3 | 4 |
| 3. Ik ben tevreden met mijn seksleven. | 1 | 2 | 3 | 4 |
| 4. Ik ben op mijn gemak met lichten aan tijdens seks. | 1 | 2 | 3 | 4 |
| 5. Ik voel mij seksueel aantrekkelijk als ik uitgekleed ben. | 1 | 2 | 3 | 4 |

**Dit is het einde van de vragenlijst.**

**Heel veel dank voor uw medewerking door het invullen van deze vragenlijst!**

Heeft u opmerkingen? Schrijf ze dan hieronder op.

…………………………………………………………………………………………………

…………………………………………………………………………………………………

…………………………………………………………………………………………………

…………………………………………………………………………………………………

…………………………………………………………………………………………………

…………………………………………………………………………………………………

………………………………………………………………………………………………
