# Supplementary material for: Burden of disease study of overweight and obesity; the societal impact in terms of cost-of-illness and health-related quality of life
Source: BMC Public Health. 2022 Jan 7;22:46. doi: 10.1186/s12889-021-12449-2 (PMC8740868; doi:10.1186/s12889-021-12449-2)
Supplement: Supplementary file 2 — Additional file 2. Costing prices. [file 12889_2021_12449_MOESM2_ESM.docx]

Additional File 2: Costing prices

| Health care service | Unit | Cost price* | Source |
| --- | --- | --- | --- |
| General practitioner | Consult | 35.69 | (21) |
| Physiotherapist | Consult | 35.69 | (21) |
| Social worker | Consult | 70.30 | (21) |
| Occupational therapist | Consult | 35.69 | (21) |
| Speech therapist | Consult | 32.45 | (21) |
| Dietitian | Consult | 28.50 | (52) |
| Homeopath or acupuncturist | Consult | 60.00 - 100 .00 | Derived from questionnaire |
| Mental health institution | Consult | 106.00 | (21) |
| Psychologist, psychotherapist,  psychiatrist - own practice | Consult | 101.67 | (21) |
| Psychologist, psychotherapist, psychiatrist - hospital | Consult | 69.22 | (21) |
| Institution for addiction  e.g. CAD** | Consult | 11.0 | (CAD, personal communication, 18 January 2021) |
| Company doctor | Consult | 115.50 | (53) |
| Hospital outpatient clinic | Consult | 98.42 | (21) |
| Day treatment hospital | Day | 298.52 | (21) |
| Other day treatment hospital  Dutch Obesity Clinic  Psychiatric institution | Day  Day | 950  326.64 | (54)  (21) |
| Emergency department | Consult | 280.13 | (21) |
| Hospital stay | Day/night | 514.84 | (21) |
| Self-help group  Weight watchers  Weight management | Consult  Consult | 18.00  29.00 | Derived from questionnaire  (55) |
| Medication costs | Prescription  Day | 6.45  Depending on medication | (21)  (56) |
| Bariatric surgery | Surgery | 10.000 | (57) |

All costs are in Euros. *All cost prices are indexed for the year 2020. **Centre for alcohol addiction and other drugs.
