# Supplementary material for: Burden of disease study of overweight and obesity; the societal impact in terms of cost-of-illness and health-related quality of life
Source: BMC Public Health. 2022 Jan 7;22:46. doi: 10.1186/s12889-021-12449-2 (PMC8740868; doi:10.1186/s12889-021-12449-2)
Supplement: Supplementary file 3 — Additional file 3. Prescribed medication list. [file 12889_2021_12449_MOESM3_ESM.docx]

Additional File 3: Prescribed medication list

| Group information | Medication | Indication |
| --- | --- | --- |
| ACE inhibitors | Enalapril  Fosinopril  Perindopril  Ramipril | Chronical kidney damage  Heart failure  Hypertension |
| Amphetamines | Methylphenidate | ADHD |
| Anti-depressive | Bupropion | Bipolar disorders  Depression  Quit smoking |
| Anticoagulant | Apixaban | Secondary prevention after TIA/CVA  Thromboembolism |
| Anticoagulants | Rivaroxaban | Coronary artery disease  Peripheral arterial disease  Thromboembolism |
| Antihistaminic | Desloratadine  Fexofenadine  Levocetirizine  Loratadine  Levocabastin | Allergic rhinitis  Chronic urticaria |
| ARB’s | Irbesartan  Losartan | Heart failure  Hypertension  Nephropathy |
| Benzodiazepine agonists | Alprazolam | Anxiety disorders |
| Benzodiazepine agonists | Zolpidem | Insomnia |
| Beta blockers | Atenolol  Bisoprolol  Metoprolol | Angina pectoris  Heart failure  Hypertension |
| Beta2-sympathicomimenticum | Cromoglicic acid  Fenoterol  Formoterol  Salbutamol  Salmeterol | Asthma  COPD |
| Biguanide | Metformin | Diabetes Mellitus type II |
| Calcineurin inhibitors | Tacrolimus | Atopic eczema  Contact eczema |
| Group information | **Medication** | **Indication** |
| Calcium salts | Calcium carbonate | Hyperphosphatemia  Osteoporosis |
| Calcium taganoists | Verapamil | Arrhythmias  Cluster headache  Hypertension |
| Colchicine | Colchicine | Gout |
| Corticosteroid | Calcipotriol  Fluticasone  Mometason | Psoriasis  COPD  Asthma  Acute rhinosinusitis  Allergic rhinitis |
| Coxib’s | Etoricoxib | Arthrosis  Gout  Nociceptive pain  Rheumatoid arthritis |
| Dihydropyridine | Amlodipine | Hypertension  Maintenance treatment of stable angina pectoris |
| Diuretic | Triamterene | Heart failure  Hypertension |
| Drugs for nicotine addiction | Varenicline | Quit smoking |
| Enamel-binding resins | Colesevelam | Hypercholesterolemia |
| Fluoroquinolones | Levofloxacin | Bacterial skin infection  Tuberculosis  Urinary tract infection |
| Heparin, LMWH’s | Nadroparin | NSTEMI-IAP ACS  STEMI-ACS  Thromboembolism |
| Insulin | Insulin glargine  Insulin glulisine | Diabetes Mellitus type I  Diabetes Mellitus type II |
| Iron preparations | Ferrous fumarate | Iron deficiency anaemia |
| Laxantia | Macrogol | Constipation |
| Mucosaprotectiva | Sucralfate | Reflux oesophagitis  Stomach complaints |
| Nitrates | Nicorandil | Maintenance treatment of stable angina pectoris |
| Group information | **Medication** | **Indication** |
| NSAID’s | Diclofenac | Migraine  Renal stone colic  Rheumatism |
| Oestrogen with progestogen, postmenopausal | Estradiol | Climacteric complaints  Osteoporosis |
| Opioid | Tramadol | Acute and chronic nociceptive pain  Arthrosis  Neuropathic pain |
| P2Y12 inhibitors | Clopidogrel | Prophylaxis of atherothrombotic thromboembolic complications  Secondary prophylaxis of atherothrombotic complications |
| Prolactin inhibitors | Quinagolide | Hyperprolactinaemia |
| Proton pump inhibitors | Esomeprazole  Omeprazole  Pantoprazole | Reflux oesophagitis  Stomach complaints  Stomach protector |
| Salicylates as aggregation inhibitors | Carbasalate calcium | Maintenance treatment of stable angina pectoris  NSTEMI-IAP ACS  STEMI-ACS  Thromboembolism |
| Salicylates as an analgesic | Acetylsalicylic acid | Hyperthermia  Lumbago  Neuralgia  Rheumatic conditions |
| Serotonin reuptake inhibitors | Citalopram  Escitalopram  Fluoxetine  Paroxetine  Sertraline | Anxiety disorders  Bipolar disorders  Depression |
| Statins | Atorvastatin  Pravastatin  Rosuvastatin  Simvastatin | Hypercholesterolemia |
| Sterol transport blocker | Ezetimibe | Hypercholesterolemia |
| Thyromimetic | Levothyroxine | Hypothyroid |
| Group information | **Medication** | **Indication** |
| Vitamin B complex | Folic acid  Hydroxocobalamin | Megaloblastic anaemia |
| Vitamin D and Analoga | Cholecalciferol | Osteoporosis  Vitamin D deficiency |
| Xanthine oxidase inhibitors | Allopurinol | Chronical kidney damage  Gout |

*Source (58)*
