# Supplementary material for: Burden of disease study of overweight and obesity; the societal impact in terms of cost-of-illness and health-related quality of life
Source: BMC Public Health. 2022 Jan 7;22:46. doi: 10.1186/s12889-021-12449-2 (PMC8740868; doi:10.1186/s12889-021-12449-2)
Supplement: Supplementary file 4 — Additional file 4. Over the counter medication list. [file 12889_2021_12449_MOESM4_ESM.docx]

Additional File 4: Over the counter medication list

| Medication | Indication |
| --- | --- |
| Calcium carbonate with magnesia carbonate | Functional stomach upset  Gastroesophageal reflux disease |
| Paracetamol | Fever and pain with flu, a cold, and after vaccination  Headache, toothache, muscle strain, and menstruation pain |
| Fish oil | Maintaining normal blood pressure  Support eyesight |
| Magnesia | Support heart and vessels  Support skeletal muscles  Convert nutrition into energy |
| L-lysine | Good for the resistance |
| Folic acid | Production red blood cells  Support functioning of the nerves |

*Source (58)*
