# Supplementary material for: Burden of disease study of overweight and obesity; the societal impact in terms of cost-of-illness and health-related quality of life
Source: BMC Public Health. 2022 Jan 7;22:46. doi: 10.1186/s12889-021-12449-2 (PMC8740868; doi:10.1186/s12889-021-12449-2)
Supplement: Supplementary file 5 — Additional file 5. Subgroup analysis of healthcare costs. [file 12889_2021_12449_MOESM5_ESM.docx]

Additional File 5. Subgroup analysis of healthcare costs

| Subgroup (N) | Costs per person (€)  Mean (SD) | Bootstrapped costs per person (€)  Mean (SD) | Bootstrapped  difference (€)  Mean (SD) | 95% CI* |
| --- | --- | --- | --- | --- |
| All | 1453.62 (3512.93) |  |  |  |
| Gender  Male (18)  Female (79) | 929.92 (1752.88)  1572.95 (3800.15) | 959.51 (404.21)  1600.59 (444.50) | 641.09 (586.32) | -507.86 – 1747.10 |
| Age  1. 19-29 (23)  2. 30 – 49 (34)  3. 50 + (40) | 1353.16 (4061.12)  1519.09 (3207.83)  1455.74 (3515.20) | 1340.93 (829.74)  1549.68 (574.46)  1486.73 (566.64) | Between  1-2 = 208.75 (1014.62)  3-2 = 62.95 (800.16)  1-3 = 145.80 (1020.53) | -2059.58 – 1920.41  -1611.67 – 1474.79  -1978.72 – 2047.54 |
| BMI  Overweight (45)  Obesity (52) | 813.38 (2930.66)  2007.68 (3891.64) | 823.28 (430.02)  1980.70 (514.68) | 1157.42 (669.75) | -184.16 – 2407.76 |
| Living situation  Living alone (29)  Living together (68) | 1647.93 (3832.42)  1370.75 (3394.22) | 1645.19 (721.78)  1379.83 (373.55) | - 265.36 (819.58) | -1973.46 – 1124.55 |
| Level of education  Low & Intermediate (43)  High (54) | 2143.38 (4163.81)  904.37 (2814.33) | 2163.15 (628.54)  902.07 (307.71) | -1261.08 (703.49) | -2719.58 – 1.69 |
| Paid work  No (14)  Yes (83) | 2264.92 (5180.67)  1316.78 (3171.95) | 2240.28 (754.83)  1317.63 (343.47) | -922.65 (808.50) | -2599.07 – 502.12 |

All costs in Euros; SD: standard deviation; CI: confidence interval; *If CI includes 0, no significant difference is found. **Significant difference.
