# Supplementary material for: Burden of disease study of overweight and obesity; the societal impact in terms of cost-of-illness and health-related quality of life
Source: BMC Public Health. 2022 Jan 7;22:46. doi: 10.1186/s12889-021-12449-2 (PMC8740868; doi:10.1186/s12889-021-12449-2)
Supplement: Supplementary file 6 — Additional file 6. Subgroup analysis of patient and family costs. [file 12889_2021_12449_MOESM6_ESM.docx]

Additional File 6. Subgroup analysis of patient and family costs

| Subgroup (N) | Costs per person (€)  Mean (SD) | Bootstrapped costs per person (€)  Mean (SD) | Bootstrapped  difference (€)  Mean (SD) | 95% CI* |
| --- | --- | --- | --- | --- |
| All | 2018.34 (2538.53) |  |  |  |
| Gender  Male (18)  Female (79) | 2189.26 (1372.05)  1980.08 (2740.98) | 2171.66 (193.65)  1992.08 (321.39) | -179.58 (370.88) | -864.32 – 638.13 |
| Age  1. 19-29 (23)  2. 30 – 49 (34)  3. 50 + (40) | 1946.21 (1309.56)  2276.68 (3833.47)  1840.23 (1538.40) | 1950.31 (263.10)  2297.71 (649.13)  1843.22 (235.03) | Between  1-2 = 347.39 (701.41)  3-2 = 454.49 (687.72)  1-3 = -107.10 (350.85) | -1891.27 – 818.42  -1985.59 – 584.18  -574.41 – 789.88 |
| BMI  Overweight (45)  Obesity (52) | 1645.18 (1344.80)  2341.63 (3215.69) | 1647.79 (202.12)  2362.99 (470.18) | 715.19 (501.07) | -88.39 – 1848.66 |
| Living situation  Living alone (29)  Living together (68) | 1886.67 (1355.98)  2074.49 (2907.63) | 1886.39 (204.69)  2075.56 (311.06) | 189.17 (372.41) | -528.66 – 938.58 |
| Level of education  Low & Intermediate (43)  High (54) | 2042.65 (3433.51)  1998.98 (1526.20) | 2033.66 (509.77)  2001.61 (166.97) | -32.04 (524.36) | -1183.25 – 767.26 |
| Paid work  No (14)  Yes (83) | 2971.93 (5887.18)  1857.49 (1366.83) | 2986.99 (879.36)  1851.44 (143.86) | - 1135.55 (881.95) | -3032.42 – 358.87 |

All costs in Euros; SD: standard deviation; CI: confidence interval; *If CI includes 0, no significant difference is found. **Significant difference.
