# Supplementary material for: Burden of disease study of overweight and obesity; the societal impact in terms of cost-of-illness and health-related quality of life
Source: BMC Public Health. 2022 Jan 7;22:46. doi: 10.1186/s12889-021-12449-2 (PMC8740868; doi:10.1186/s12889-021-12449-2)
Supplement: Supplementary file 7 — Additional file 7. Subgroup analysis of other costs. [file 12889_2021_12449_MOESM7_ESM.docx]

Additional File 7. Subgroup analysis of other costs

| Subgroup (N) | Costs per person (€)  Mean (SD) | Bootstrapped costs per person (€)  Mean (SD) | Bootstrapped difference (€)  Mean (SD) | 95% CI* |
| --- | --- | --- | --- | --- |
| All | 2259.37 (6141.23) |  |  |  |
| Gender  Male (18)  Female (79) | 2717.12 (8931.13)  2155.07 (5382.76) | 2748.47 (2082.77)  2137.37 (583.61) | - 611.10 (2147.49) | -5378.88 – 2528.20 |
| Age  1. 19-29 (23)  2. 30 – 49 (34)  3. 50 + (40) | 1222.64 (2865.37)  4073.09 (9627.64)  1313.83 (2314.09) | 1229.24 (615.62)  4105.32 (1606.60)  1320.08 (363.70) | Between  1-2 = 2876.08 (1731.80)  3-2 = 2785.24 (1640.27)  1-3 = 90.84 (731.15) | -6569.42 – 140.76  -6283.05 – 44.16  -1340.65 – 1492.70 |
| BMI  Overweight (45)  Obesity (52) | 997.66 (2345.90)  3351.24 (7976.49) | 999.77 (345.50)  3397.30 (1093.14) | 2397.53 (1152.26) ** | 377.14 – 4739.04** |
| Living situation  Living alone (29)  Living together (68) | 3288.98 (7449.74)  1820.27 (5494.64) | 3305.26 (1096.74)  1792.10 (674.97) | - 1513.08 (1305.52) | -4368.28 – 800.60 |
| Level of education  Low & Intermediate (43)  High (54) | 3544.48 (8617.12)  1236.04 (2657.67) | 3665.84 (1280.85)  1231.71 (278.12) | -2434.13 (1314.29)** | -5186.15 – -228.22** |
| Paid work  No (14)  Yes (83) | 3038.03 (10476.66)  2128.03 (5160.61) | 3064.79 (1513.47)  2121.47 (560.83) | -943.32 (1621.15) | -4203.12 – 2108.51 |

All costs in Euros; SD: standard deviation; CI: confidence interval; *If CI includes 0, no significant difference is found. **Significant difference.
