# Supplementary material for: Burden of disease study of overweight and obesity; the societal impact in terms of cost-of-illness and health-related quality of life
Source: BMC Public Health. 2022 Jan 7;22:46. doi: 10.1186/s12889-021-12449-2 (PMC8740868; doi:10.1186/s12889-021-12449-2)
Supplement: Supplementary file 8 — Additional file 8. Subgroup analysis of total societal costs. [file 12889_2021_12449_MOESM8_ESM.docx]

Additional File 8. Subgroup analysis of total societal costs

| Subgroup (N) | Costs per person (€)  Mean (SD) | Bootstrapped costs per person (€)  Mean (SD) | Bootstrapped difference (€)  Mean (SD) | 95% CI* |
| --- | --- | --- | --- | --- |
| All | 5731.33 (8238.70) |  |  |  |
| Gender  Male (18)  Female (79) | 5833.30 (11317.08)  5708.10 (7458.07) | 5903.59 (1738.99)  5736.86 (804.65) | -166.73 (1922.39) | -4186.45 – 3131.24 |
| Age  1. 19-29 (23)  2. 30 – 49 (34)  3. 50 + (40) | 4522.01 (5614.39)  7868.86 (11980.96)  4609.79 (4658.61) | 4503.84 (1114.25)  7781.73 (2064.50)  4593.53 (720.87) | Between  1-2 = 3277.89 (2388.21)  3-2 = 3188.20 (2126.77)  1-3 = 89.69 (1373.70) | -8298.30 – 1182.54  -7698.65 – 750.30  -2552.05 – 2670.81 |
| BMI  Overweight (45)  Obesity (52) | 3456.22 (4103.90)  7700.18 (10233.13) | 3465.70 (622.29)  7680.50 (1422.92) | 4214.81 (1574.45)** | 1349.57 – 7447.27** |
| Living situation  Living alone (29)  Living together (68) | 6823.59 (9779.01)  5265.52 (7520.26) | 6816.21 (1438.32)  5277.08 (777.98) | -1539.13 (1648.92) | -4883.67 – 1354.61 |
| Level of education  Low & Intermediate (43)  High (54) | 7730.51 (11026.45)  4139.39 (4557.66) | 7803.93 (1695.25)  4143.30 (492.21) | -3660.62 (1799.31)** | -7200.80 – -385.95** |
| Paid work  No (14)  Yes (83) | 8274.87 (11869.52)  5302.30 (7472.50) | 8304.68 (1780.08)  5371.37 (799.77) | -2933.31 (1995.07) | -7127.61 – 894.32 |

All costs in Euros; SD: standard deviation; CI: confidence interval; *If CI includes 0, no significant difference is found. **Significant difference.
