# Supplementary material for: Burden of disease study of overweight and obesity; the societal impact in terms of cost-of-illness and health-related quality of life
Source: BMC Public Health. 2022 Jan 7;22:46. doi: 10.1186/s12889-021-12449-2 (PMC8740868; doi:10.1186/s12889-021-12449-2)
Supplement: Supplementary file 9 — Additional file 9. Subgroup analysis utility score derived from the five-dimensional, five-level EuroQol. [file 12889_2021_12449_MOESM9_ESM.docx]

Additional File 9. Subgroup analysis utility score derived from the five-dimensional, five-level EuroQol.

| Subgroup (N) | Utility score |  |  |  |
| --- | --- | --- | --- | --- |
|  | Min | Max | Mean (SD) | Median |
| All | 0.29 | 1.00 | 0.81 (0.18) | 0.83 |
| Gender  Male (18)  Female (79) | 0.49  0.29 | 1.00  1.00 | 0.83 (0.15)  0.80 (0.18) | 0.84  0.83 |
| Age  1. 19-29 (23)  2. 30 – 49 (34)  3. 50 + (40) | 0.45  0.29  0.32 | 1.00  1.00  1.00 | a**  0.87 (0.19)  0.79 (0.19)  0.79 (0.15) | 1.00  0.83  0.82 |
| BMI  Overweight (45)  Obesity (52) | 0.41  0.29 | 1.00  1.00 | **  0.86 (0.14)  0.77 (0.19) | 0.87  0.81 |
| Living situation  Living alone (29)  Living together (68) | 0.32  0.29 | 1.00  1.00 | 0.77 (0.20)  0.83 (0.16) | 0.81  0.83 |
| Level of education  Low & intermediate (43)  High (54) | 0.29  0.32 | 1.00  1.00 | 0.79 (0.18)  0.83 (0.17) | 0.82  0.84 |
| Paid work  No (14)  Yes (83) | 0.29  0.30 | 1.00  1.00 | 0.83 (0.16)  0.71 (0.22) | 0.83  0.78 |

SD: standard deviation. **Significant difference. a**Significant difference between group 1-2 and 1-3.
