# Supplementary material for: Burden of disease study of overweight and obesity; the societal impact in terms of cost-of-illness and health-related quality of life
Source: BMC Public Health. 2022 Jan 7;22:46. doi: 10.1186/s12889-021-12449-2 (PMC8740868; doi:10.1186/s12889-021-12449-2)
Supplement: Supplementary file 10 — Additional file 10. Subgroup analysis Rasch-score derived from BODY-Q, scale of psychological well-being. Subgroup analysis Rasch-score derived from BODY-Q, scale of social well-being. Subgroup analysis Rasch-score derived from BODY-Q, scale of body image. Subgroup analysis Rasch-score derived from BODY-Q, scale of physical well-being. Subgroup analysis Rasch-score derived from BODY-Q, scale of sexual well-being. [file 12889_2021_12449_MOESM10_ESM.zip › Additional File 10.1.docx]

Additional File 10.1. Subgroup analysis Rasch-score derived from BODY-Q, scale of psychological well-being.

| Psychological well-being |  |  |  |  |
| --- | --- | --- | --- | --- |
| Subgroup (N) |  |  |  |  |
|  | Min | Max | Mean (SD) | Median |
| All (97) | 0.00 | 100.00 | 60.55 (21.02) | 62.00 |
| Gender  Male (18)  Female (79) | 18.00  0.00 | 100.00  100.00 | 61.83 (23.70)  60.25 (20.52) | 63.50  62.00 |
| Age  19-29 (23)  30 – 49 (34)  50 + (40) | 32.00  0.00  18.00 | 100.00  100.00  100.00 | 61.74 (17.18)  56.21 (23.90)  63.55 (20.29) | 65.00  57.50  62.00 |
| BMI  Overweight (45)  Obese (52) | 18.00  0.00 | 100.00  100.00 | 64.76 (20.73)  56.90 (20.78) | 65.00  58.50 |
| Living situation  Living alone (29)  Living together (68) | 18.00  0.00 | 100.00  100.00 | 57.14 (19.95)  62.00 (21.44) | 57.00  62.00 |
| Level of education  Low & intermediate (43)  High (54) | 0.00  32.00 | 100.00  100.00 | **  55.19 (23.80)  64.81 (17.60) | 55.00  65.00 |
| Paid work  No (14)  Yes (83) | 0.00  13.00 | 92.00  100.00 | 52.29 (24.13)  61.94 (20.28) | 52.00  62.00 |

SD: standard deviation. **Significant difference.
