# Supplementary material for: Burden of disease study of overweight and obesity; the societal impact in terms of cost-of-illness and health-related quality of life
Source: BMC Public Health. 2022 Jan 7;22:46. doi: 10.1186/s12889-021-12449-2 (PMC8740868; doi:10.1186/s12889-021-12449-2)
Supplement: Supplementary file 10 — Additional file 10. Subgroup analysis Rasch-score derived from BODY-Q, scale of psychological well-being. Subgroup analysis Rasch-score derived from BODY-Q, scale of social well-being. Subgroup analysis Rasch-score derived from BODY-Q, scale of body image. Subgroup analysis Rasch-score derived from BODY-Q, scale of physical well-being. Subgroup analysis Rasch-score derived from BODY-Q, scale of sexual well-being. [file 12889_2021_12449_MOESM10_ESM.zip › Additional File 10.2.docx]

Additional File 10.2. Subgroup analysis Rasch-score derived from BODY-Q, scale of social well-being.

| Social well-being |  |  |  |  |
| --- | --- | --- | --- | --- |
| Subgroup (N) |  |  |  |  |
|  | Min | Max | Mean (SD) | Median |
| All (97) | 0.00 | 100.00 | 63.13 (19.63) | 62.00 |
| Gender  Male (18)  Female (79) | 25.00  0.00 | 100.00  100.00 | 60.50 (20.06)  63.73 (19.61) | 63.50  60.00 |
| Age  19-29 (23)  30 – 49 (34)  50 + (40) | 44.00  0.00  38.00 | 100.00  100.00  100.00 | 65.26 (14.27)  60.15 (24.58)  64.45 (17.61) | 62.00  58.50  63.50 |
| BMI  Overweight (45)  Obese (52) | 34.00  0.00 | 100.00  100.00 | 66.36 (18.34)  60.35 (20.44) | 62.00  61.00 |
| Living situation  Living alone (29)  Living together (68) | 25.00  0.00 | 100.00  100.00 | 34.48 (24.66)  64.44 (19.91) | 57.00  65.00 |
| Level of education  Low & intermediate (43)  High (54) | 0.00  34.00 | 100.00  100.00 | **  58.65 (19.48)  66.70 (19.18) | 60.00  65.00 |
| Paid work  No (14)  Yes (83) | 0.00  25.00 | 81.00  100.00 | **  52.71 (21.77)  64.89 (18.82) | 56.00  62.00 |

SD: standard deviation. **Significant difference.
