# Supplementary material for: Burden of disease study of overweight and obesity; the societal impact in terms of cost-of-illness and health-related quality of life
Source: BMC Public Health. 2022 Jan 7;22:46. doi: 10.1186/s12889-021-12449-2 (PMC8740868; doi:10.1186/s12889-021-12449-2)
Supplement: Supplementary file 10 — Additional file 10. Subgroup analysis Rasch-score derived from BODY-Q, scale of psychological well-being. Subgroup analysis Rasch-score derived from BODY-Q, scale of social well-being. Subgroup analysis Rasch-score derived from BODY-Q, scale of body image. Subgroup analysis Rasch-score derived from BODY-Q, scale of physical well-being. Subgroup analysis Rasch-score derived from BODY-Q, scale of sexual well-being. [file 12889_2021_12449_MOESM10_ESM.zip › Additional File 10.3.docx]

Additional File 10.3. Subgroup analysis Rasch-score derived from BODY-Q, scale of body image.

| Body image |  |  |  |  |
| --- | --- | --- | --- | --- |
| Subgroup (N) |  |  |  |  |
|  | Min | Max | Mean (SD) | Median |
| All (97) | 0.00 | 92.00 | 36.37 (24.59) | 38.00 |
| Gender  Male (18)  Female (79) | 0.00  0.00 | 92.00  92.00 | 40.89 (24.30)  35.34 (24.69) | 44.00  38.00 |
| Age  19-29 (23)  30 – 49 (34)  50 + (40) | 0.00  0.00  0.00 | 92.00  74.00  92.00 | a**  41.87 (24.05)  30.24 (23.58)  38.43 (25.24) | 44.00  35.00  38.00 |
| BMI  Overweight (45)  Obese (52) | 0.00  0.00 | 92.00  92.00 | **  43.98 (22.81)  29.79 (24.37) | 44.00  31.00 |
| Living situation  Living alone (29)  Living together (68) | 0.00  0.00 | 92.00  92.00 | 34.48 (24.66)  37.18 (24.70) | 38.00  39.50 |
| Level of education  Low & intermediate (43)  High (54) | 0.00  0.00 | 85.00  92.00 | 30.49 (25.59)  41.06 (22.93) | 31.00  41.00 |
| Paid work  No (14)  Yes (83) | 0.00  0.00 | 92.00  92.00 | 28.36 (29.53)  37.72 (23.60) | 26.50  41.00 |

SD: standard deviation. **Significant difference. a**Significant difference between group 1-2.
