# Supplementary material for: Burden of disease study of overweight and obesity; the societal impact in terms of cost-of-illness and health-related quality of life
Source: BMC Public Health. 2022 Jan 7;22:46. doi: 10.1186/s12889-021-12449-2 (PMC8740868; doi:10.1186/s12889-021-12449-2)
Supplement: Supplementary file 10 — Additional file 10. Subgroup analysis Rasch-score derived from BODY-Q, scale of psychological well-being. Subgroup analysis Rasch-score derived from BODY-Q, scale of social well-being. Subgroup analysis Rasch-score derived from BODY-Q, scale of body image. Subgroup analysis Rasch-score derived from BODY-Q, scale of physical well-being. Subgroup analysis Rasch-score derived from BODY-Q, scale of sexual well-being. [file 12889_2021_12449_MOESM10_ESM.zip › Additional File 10.4.docx]

Additional File 10.4. Subgroup analysis Rasch-score derived from BODY-Q, scale of physical well-being.

| Physical well-being |  |  |  |  |
| --- | --- | --- | --- | --- |
| Subgroup (N) |  |  |  |  |
|  | Min | Max | Mean (SD) | Median |
| All (97) | 15.00 | 100.00 | 72.93 (21.01) | 71.00 |
| Gender  Male (18)  Female (79) | 47.00  15.00 | 100.00  100.00 | 76.22 (16.38)  72.18 (21.95) | 73.50  71.00 |
| Age  19-29 (23)  30 – 49 (34)  50 + (40) | 26.00  15.00  36.00 | 100.00  100.00  100.00 | a**  82.00 (20.89)  71.62 (24.03)  68.83 (16.86) | 90.00  71.00  68.50 |
| BMI  Overweight (45)  Obese (52) | 42.00  15.00 | 100.00  100.00 | **  79.69 (18.51)  67.08 (21.45) | 82.00  66.00 |
| Living situation  Living alone (29)  Living together (68) | 39.00  15.00 | 100.00  100.00 | 75.52 (20.55)  71.82 (21.26) | 82.00  71.00 |
| Level of education  Low & intermediate (43)  High (54) | 15.00  26.00 | 100.00  100.00 | 68.44 (20.65)  76.50 (20.79) | 66.00  82.00 |
| Paid work  No (14)  Yes (83) | 15.00  26.00 | 100.00  100.00 | 64.37 (25.89)  74.37 (19.89) | 71.00  71.00 |

SD: standard deviation. **Significant difference. a**Significant difference between group 1-3.
