# Supplementary material for: Burden of disease study of overweight and obesity; the societal impact in terms of cost-of-illness and health-related quality of life
Source: BMC Public Health. 2022 Jan 7;22:46. doi: 10.1186/s12889-021-12449-2 (PMC8740868; doi:10.1186/s12889-021-12449-2)
Supplement: Supplementary file 10 — Additional file 10. Subgroup analysis Rasch-score derived from BODY-Q, scale of psychological well-being. Subgroup analysis Rasch-score derived from BODY-Q, scale of social well-being. Subgroup analysis Rasch-score derived from BODY-Q, scale of body image. Subgroup analysis Rasch-score derived from BODY-Q, scale of physical well-being. Subgroup analysis Rasch-score derived from BODY-Q, scale of sexual well-being. [file 12889_2021_12449_MOESM10_ESM.zip › Additional File 10.5.docx]

Additional File 10.5. Subgroup analysis Rasch-score derived from BODY-Q, scale of sexual well-being.

| Sexual well-being |  |  |  |  |
| --- | --- | --- | --- | --- |
| Subgroup (N) |  |  |  |  |
|  | Min | Max | Mean (SD) | Median |
| All (74) | 0.00 | 100.00 | 58.69 (22.33) | 58.00 |
| Gender  Male (13)  Female (61) | 26.00  0.00 | 86.00  100.00 | 58.69 (19.23)  58.69 (23.08) | 63.00  54.00 |
| Age  19-29 (23)  30 – 49 (34)  50 + (40) | 35.00  26.00  0.00 | 100.00  100.00  100.00 | a**  68.21 (19.74)  51.68 (18.49)  59.26 (25.62) | 68.00  49.00  63.00 |
| BMI  Overweight (31)  Obese (43) | 26.00  0.00 | 100.00  100.00 | **  64.87 (19.89)  54.23 (23.15) | 63.00  51.00 |
| Living situation  Living alone (19)  Living together (55) | 0.00  26.00 | 100.00  100.00 | 53.00 (24.84)  60.65 (21.29) | 47.00  58.00 |
| Level of education  Low & intermediate (33)  High (41) | 0.00  26.00 | 100.00  100.00 | 55.12 (24.99)  61.56 (19.79) | 47.00  63.00 |
| Paid work  No (13)  Yes (61) | 0.00  26.00 | 75.00  100.00 | **  41.85 (18.43)  62.28 (21.55) | 39.00  63.00 |

SD: standard deviation. **Significant difference. a**Significant difference between group 1-2.
